# Design and Validation of DNA Libraries for Multiplexing Proximity Ligation Assays

**DOI:** 10.1371/journal.pone.0112629

**Published:** 2014-11-11

**Authors:** Nicolas Gobet, Simon Ketterer, Matthias Meier

**Affiliations:** 1 IMTEK, Department of Microsystems Engineering, Microfluidic and Biological Engineering, University of Freiburg, Freiburg, Germany; 2 BIOSS-Centre for Signalling Studies, University of Freiburg, Freiburg, Germany; Imperial College London, United Kingdom

## Abstract

Here, we present an *in silico*, analytical procedure for designing and testing orthogonal DNA templates for multiplexing of the proximity ligation assay (PLA). PLA is a technology for the detection of protein interactions, post-translational modifications, and protein concentrations. To enable multiplexing of the PLA, the target information of antibodies was encoded within the DNA template of a PLA, where each template comprised four single-stranded DNA molecules. Our DNA design procedure followed the principles of minimizing the free energy of DNA cross-hybridization. To validate the functionality, orthogonality, and efficiency of the constructed template libraries, we developed a high-throughput solid-phase rolling-circle amplification assay and solid-phase PLA on a microfluidic platform. Upon integration on a microfluidic chip, 640 miniaturized pull-down assays for oligonucleotides or antibodies could be performed in parallel together with steps of DNA ligation, isothermal amplification, and detection under controlled microenvironments. From a large computed PLA template library, we randomly selected 10 template sets and tested all DNA combinations for cross-reactivity in the presence and absence of antibodies. By using the microfluidic chip application, we determined rapidly the false-positive rate of the design procedure, which was less than 1%. The combined theoretical and experimental procedure is applicable for high-throughput PLA studies on a microfluidic chip.

## Introduction

**Figure 1:**
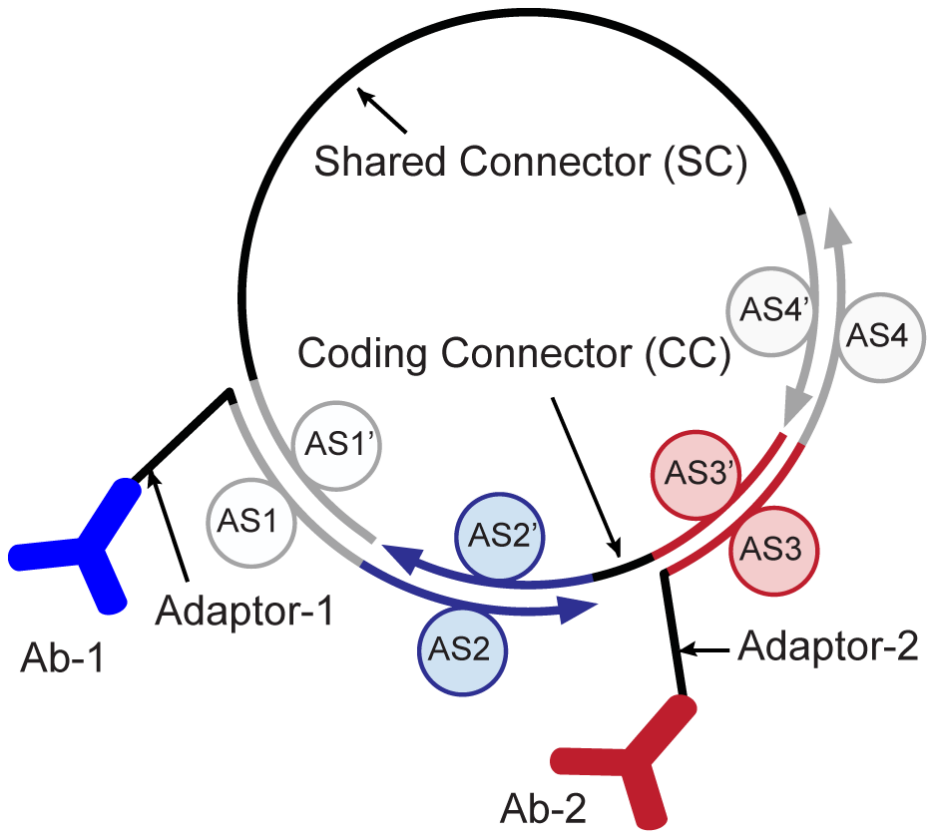
DNA configuration of a proximity ligation assay (PLA) template for multiplexing. One PLA template comprises two adaptor and connector DNA strands. The adaptor strands are conjugated to an antibody (Ab) and each exhibits two annealing sequences (ASs). The connector strands exhibit the complementary sequences of the two AS sites located on different adaptors (AS′). In cases where the two antibodies and, consequently, the adaptors are in close proximity, the connector strands with matching AS sites can hybridize and form a circular DNA ring. Upon ligation, the circular DNA can be amplified by rolling-circle amplification (RCA). Multiplexing of the PLA is enabled by encoding the target information of the antibody in the AS region of the adaptor for one connector. Arrowheads on all DNA strands mark the 3′ end of the oligonucleotides.

Multiplexing of analytical assay technologies is a major challenge in protein analytics [1]. Western blots, enzyme-linked immunosorbent assays (ELISA), mass spectrometry, or miniaturized immuno assays on microfluidic chip platforms are standard applications in protein analytics [2]–[6]. The technologies differ strongly in their capability of multiplexing, ranging from purely sequential operations to highly parallel processing on microfluidic chips. Data sets acquired with these technologies, however, show often a negative mutual dependence between throughput and specificity, as well as sensitivity [7]. More recently, the proximity ligation assay (PLA) was added to the toolbox of protein analytics [8]. The PLA technology allows the detection of protein concentrations, modifications, and/or posttranslational modifications. Within a basic configuration, two antibodies are labeled with oligonucleotides. Upon binding of the antibodies to their targets, the proximity of the oligonucleotide labels is tested by a hybridization reaction. For this step, two additional DNA connector strands are used to bridge the antibody oligonucleotide labels. Ligation of the connectors leads to the formation of a circular DNA fragment, which serves as a template for isothermal rolling circle amplification (RCA) [9]. The DNA template configuration of a PLA experiment is shown in Figure 1. Variations of the basic PLA template configuration and assay have been developed for protein analytics in solutions [10], solid supports [11], [12], cells [13], and tissues [14]. Translation of the proximity information from the protein to the DNA level and utilization of DNA amplification methods for signal enhancement result in a protein assay with high sensitivity, down to single molecules [15], and high specificity. A caveat of the assay is the complexity of the system, including labeling of the affinity reagents, and multiple reactions steps paired with changing reaction conditions and longer incubation times.

Through multiplexing of the PLA, the complexity of the assay workflow can be overcome. Several multiplexing approaches of the PLA have been reported with the common strategy to encode the target specificity of the antibody within the oligonucleotide label [16]. Either next-generation sequencing [14] or multicolor fluorescence detection methods [17] are used for parallel decoding of proximity signals. In the latter case, detection probe sequences are included in the DNA configuration of the PLA template. An alternative method to multiplex PLA reactions is to use the microfluidic chip technology [18], where analytical solutions or cells are spatially separated. Parallel processing of separated PLA reactions has the advantage of allowing the use of the same template configuration for different targets.

For all multiplexed PLA approaches, orthogonal DNA templates have to be designed [19], [20]. The combinatorial space for encoding proximity signals in the PLA template is large. Manual designs of PLA templates are prone to error because of the multiplicity of possible cross-hybridization events between all DNA strands in a PLA template library.

Here, we have developed a procedure for designing orthogonal PLA templates to enable and customize multiplexing of PLAs. The design procedure implements a minimal free energy of hybridization approach between mismatched DNA strands forming a PLA templates in a composite. We exemplary generated a larger library of PLA templates and tested the orthogonality of 10 randomly selected PLA templates. For this, a rapid oligonucleotide pull-down assay in conjugation with a RCA reaction was integrated on a microfluidic chip. Cross-reactions between DNA elements in the library were determined solely on the basis of the DNA sequence. In a following step, PLA templates proven to be orthogonal on the DNA basis were tested again for orthogonality within a solid-phase PLA (spPLA) on the chip. With this we investigated the influence of the antibodies on the DNA assembling efficiency. Upon miniaturization and parallelization of both test systems within a microfluidic chip platform [5], [21], we were able to screen all combinatorial possibilities of the DNA components in the multiplexing library. Furthermore, the chip results were used to calculate false-positive rates of the *in silico* design procedure.

## Experimental Procedures

### Software implementation

The PLA oligonucleotide design program was written in C and optimized for parallel computing on a Unix cluster with OpenMP. RNAfold and RNAplex from the ViennaRNA package [22] were integrated to calculate minimum folding and interaction energies, respectively. The source code of the software is given in File S1.

### Chip assembling

All oligonucleotides were purchased from Sigma-Aldrich (Taufkirchen, Germany). Adaptor and connector oligonucleotides were biotinylated or phosphorylated at the 5′ end, respectively. Binary combinations of adaptor or connector oligonucleotides at a concentration of 10 µM were dissolved in a buffer containing 22.5 mM sodium citrate, 0.22 M sodium chloride, and 0.5 mg/mL bovine serum albumin (BSA). Adaptor solutions were spotted onto epoxy-coated microscope slides at room temperature; the humidity for the print was adjusted to 70%. An OmniGrid Micro (Digilab, MA) contact printer with a custom-made print-head holding silicon pins (Parallel Synthesis Technologies, CA) was used for all prints. Dried DNA microarrays were aligned and bound to the microfluidic polydimethylsiloxane (PDMS) chip overnight at 80°C. Multilayer PDMS chips were fabricated following standard protocols as published [23]. The fluidic chip interface was controlled with Matlab (Mathworks, MA).

### Antibody DNA conjugation

Adapter oligonucleotides were conjugated to vascular endothelial growth factor (VEGF) IgG antibodies as previously described [15]. In detail, antibodies were activated by 30× molar excess (Thermo Scientific, Germany) of sulfosuccinimidyl-(4-N-maleimidomethyl)cyclohexane-1-carboxylate (sulfo-SMCC) (Sigma Aldrich, Germany) for 2 h at room temperature in 1× phosphate-buffered saline (PBS), containing 5 mM EDTA (pH 7.35). Sulfo-SMCC-activated IgG was purified on Zeba spin desalting columns, 7K MWCO (Perbio Science, France). SH-adapters were reduced at 14 mM in 30 mM dithiothreitol for 1 h at 37°C before purification on Illustra MicroSpin G-25 columns. The reduced adaptors were immediately added to sulfo-SMCC-activated IgG in 3× molar excess. The product was dialyzed overnight at 4°C in Slide-A-Lyzer MINI dialysis devices, 7K MWCO (Thermo Fisher, Germany). The final concentration of the DNA-conjugated antibodies was determined with a BCA protein assay (Novagen, MA).

### On-chip solid-phase RCA and PLA

The workflow, conditions, and chemical reagents for the integrated microfluidic solid-phase RCA (spRCA) and PLA reactions are given in detail in File S2 and S3.

### Image acquisition and signal processing

Images were acquired using a Zeiss Axio Observer (Zeiss, Jena) inverted fluorescence microscope with a 20× objective and 1.6× tube lens. Two fluorescence images were acquired for each reaction chamber using the filter sets 38HE and 43HE (Zeiss, Jena) for the fluorophores 6-carboxyfluorescein (6-FAM) and rhodamine-X, respectively. Images were analyzed using the Matlab Image Processing Toolbox (Mathworks). We extracted the median fluorescence intensity of the 6-FAM probe for the pull-down area within each unit cell. A local background signal was subtracted from all values. For this, an area enclosing the pull-down area and of twice the size of the pull-down area was evaluated.

## Results

The PLA combines two classes of biomolecules, i.e., antibodies for target detection and DNA for signal amplification. Both biomolecule classes have to be orthogonal to allow multiplexing in one composite. To optimize the DNA elements of PLA templates, we first investigated the DNA specificity in the absence of antibodies to avoid overlapping effects from the two different molecule groups. In a second step, only orthogonal PLA templates were tested within a spPLA reaction in the presence of antibodies.

Figure 1 shows the configuration of the PLA template design for multiplexing with two adaptor and connector strands. Adaptors contain two annealing sequences (ASs), one for each connector strand. In turn, the connectors contain the reverse complement sequences to the annealing sites of the adaptors (AS′). A PLA template library for multiplexing can be generated by alternating the AS sites on both adaptors for one connector, which hereafter is referred to as the coding connector (CC). This PLA template configuration allows sharing of the second connector between all PLA templates. For the detection of PLA events, we included a sequence for a fluorescence probe molecule in the shared connector (SC). The number of DNA strands required to develop a PLA template library with the above configuration and for n targets can be calculated as follows: (i) one constant SC, (ii) n adaptors, and (iii) (n−1)×n/2 CC strands are required if all possible pairwise interactions between the adaptors are tested. The latter equation for the CC is derived from the Gauss summation formula.

### In silico generation of PLA templates

Sequence boundaries for AS sites are defined on the basis of biochemical restraints of the PLA. Melting temperatures, *T*_m_, between matched adaptor and connector DNA are desired to be in the order of the working temperature of the T4 ligase, which is 37°C. Long annealing sites with higher *T*_m_ values will tolerate base pair mismatches between adaptor and connector strands at the working temperature of the ligase. Short annealing sites with *T*_m_ lower than the ligase working temperature will decrease the PLA efficiency and exhibit a smaller sequence distances within a multiplex library. For 10^7^ randomly generated DNA sequences with a lengths of 10, 11, or 12 nucleotides (nt), we calculated a mean *T*_m_ value of 31°C, 34.5°C, and 40.5°C, respectively. For the calculation of the *T*_m_ values a sodium, magnesium, and DNA concentration of 50 mM, 1 mM, 0.1 µm was used respectively. On the basis of this result, we decided to generate AS sites with a length of 11 nt.

**Figure 2:**
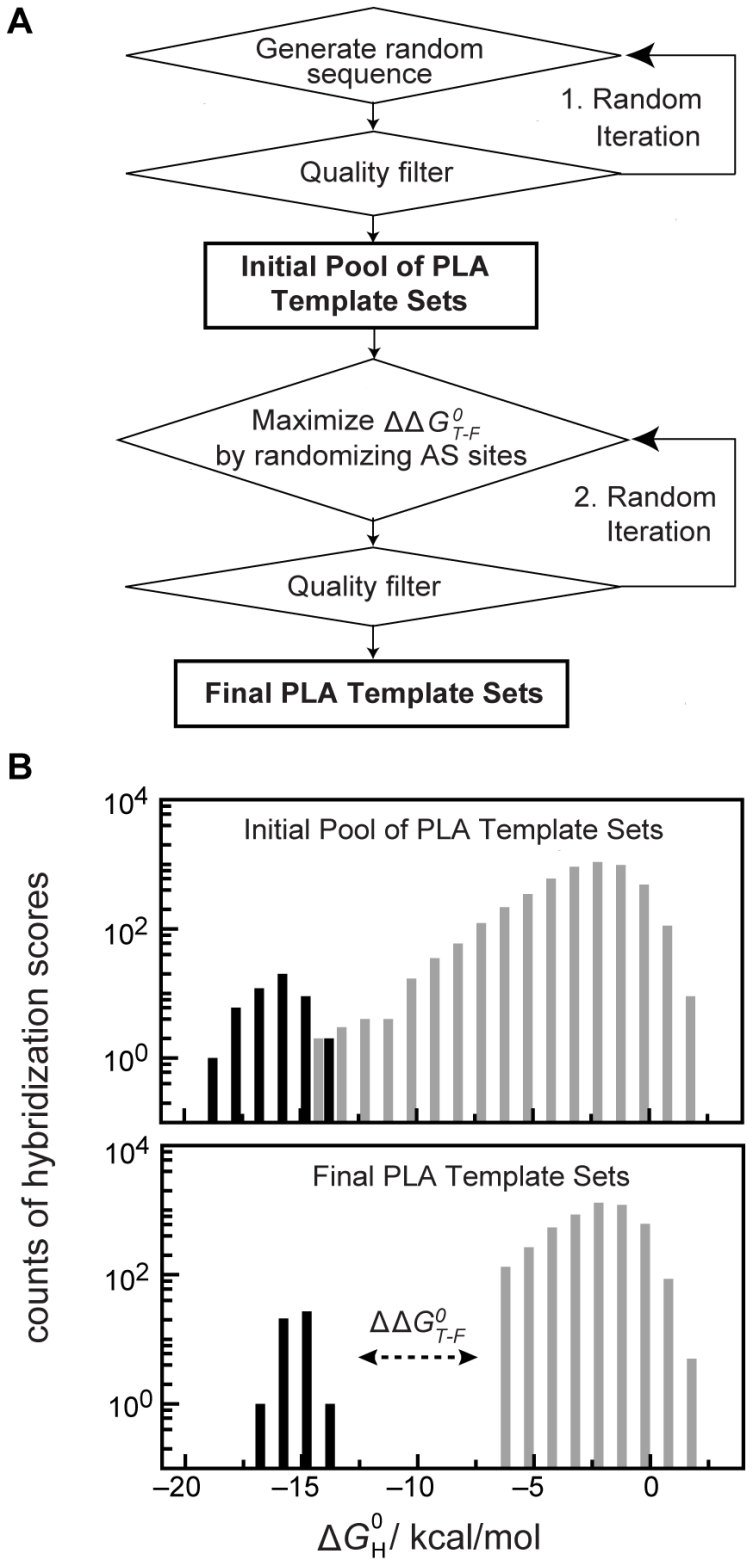
*In silico* PLA template design procedure. (A) Computational flow chart to obtain orthogonal proximity ligation assay (PLA) templates. The process is divided into two randomization steps. In the first step, the annealing sequence pairs (AS/AS′) are generated and subjected to GC content, nucleotide bias, secondary structure, and cross-hybridization filters. The second randomization step maximizes the differences in the hybridization energy between the largest observed value for a matched AS/AS′ pair and the lowest observed value for a matched AS/AS′ pair (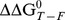) in the composite. (B) Upper and lower bar charts show the DNA hybridization energies between all AS sites within the 24 PLA templates after the first and second selection and randomization steps, respectively. Grey and black bars denote hybridization events between unmatched and matched AS sites within the generated PLA template library.

The *in silico* procedure for designing PLA templates is divided into two randomization processes. In the first step, all single-stranded DNA sequences are generated, and in the second step, their orthogonality is maximized. Constant DNA sequences shared between all PLA templates are defined at the beginning and are based on previously published designs [12]. The AS and AS′ sites for adaptor and connector strands are randomly generated and subjected to a quality filter. Only AS/AS′ sequence pairs with a GC content of 50±5%, a single nucleotide bias of 50±10%, and without secondary DNA structure elements could pass the filter. For prediction of structure elements in the AS sites we used the default parameters of DNAfold [22]. Newly generated AS sites were additionally tested for cross-hybridization to already selected sequences. Here, the cross-hybridization energies to any sequence in the selected pool must be lower than 40% of the hybridization energy between matched AS/AS′ sites. The cut-off value of 40% for the hybridization energy was empirically chosen. Lower cut-off values led to a strong increase of the computation time. Hybridization energies were calculated using RNAplex [24]. Within the second randomization process, the orthogonality between the sequences in the initial pool of PLA templates was maximized. To achieve this, the free energies of hybridization events of mismatched DNA strands among the sequences were globally minimized by iterative random mutation of the AS sites. The same quality filter used in the first randomization process was applied. The salt and temperature conditions within the RNAplex algorithms were set to the default values. The flow chart of the design procedure is given in Figure 2A. The mutation process was continued until the minimum free energy of all cross-hybridization events was found.

The free energy values of all combinatorial hybridization events between AS sites, 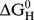, of 24 PLA templates after the first and second randomization steps are shown in Figure 2B. 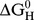 values of matched (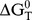) and mismatched (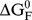) AS sites within the initial pool of randomly generated AS sites varied between −19 to −14 kcal/mol and −14 to 2 kcal/mol, respectively. The stringed sequence filter function in step one of the design procedure alone could not create an energy difference between the highest 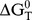 value of the matched and the lowest 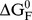 value of the mismatched AS site hybridizations (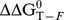). After the second randomization process of the AS sites, a 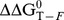 value of 8 kcal/mol was achieved. This equals the energy of 2–3 base pair mismatches within a hybridization reaction of an AS/AS′ pair [25]. The sequences of all generated PLA templates are given in Table S1. To confirm the orthogonality of the generated PLA templates, we developed a spRCA on the DNA of PLA templates and a spPLA test system on a microfluidic chip.

### The microfluidic framework

**Figure 3:**
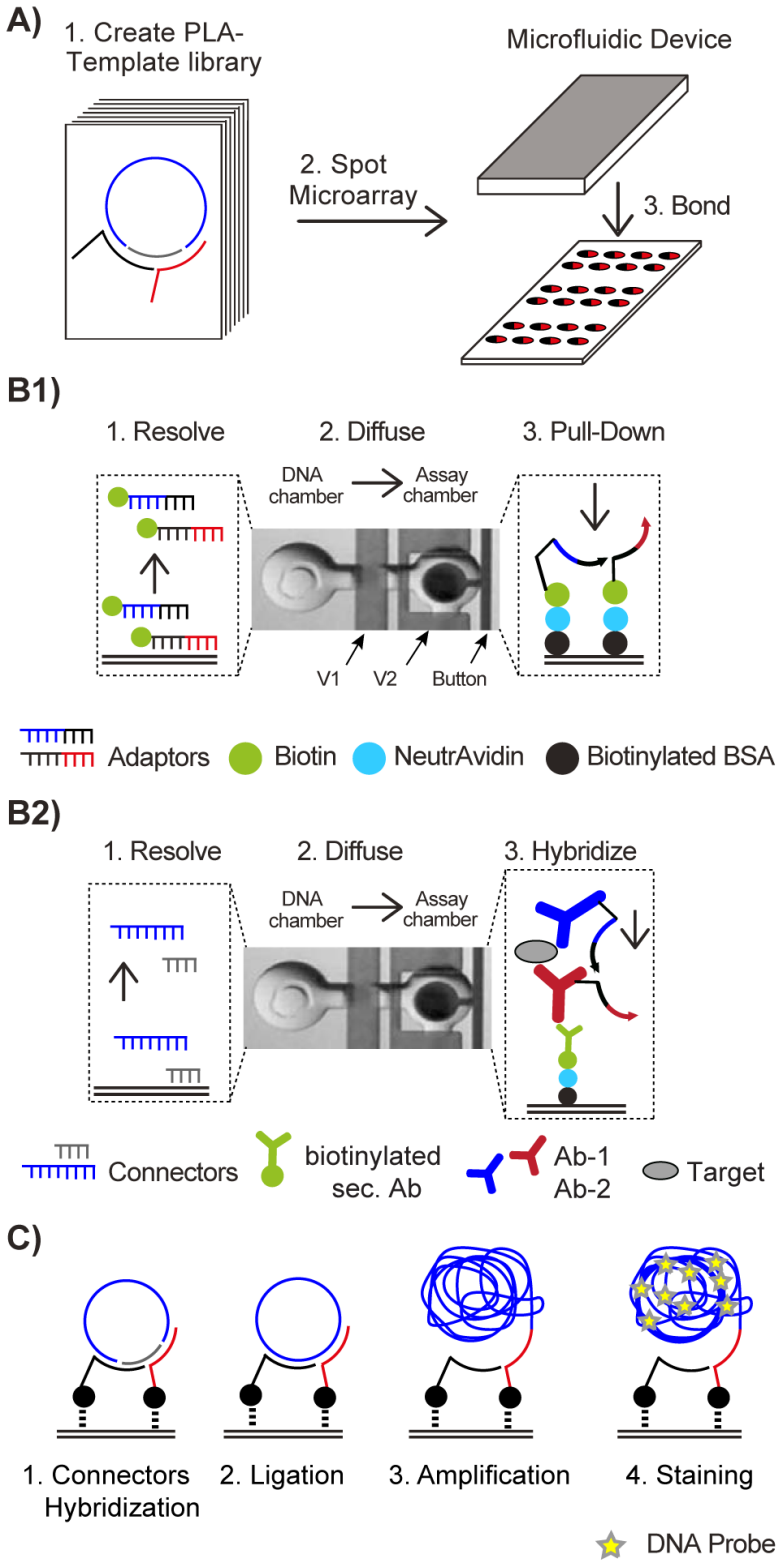
Microfluidic chip integration of a solid-phase rolling-circle amplification (RCA) and proximity ligation assay (PLA) system for testing PLA templates. (A) Pairs of either adaptor or connector strands from a PLA template library are plotted on an epoxy-coated glass slide and aligned to a polydimethylsiloxane (PDMS)-based microfluidic chip. (B1) DNA-only PLA test system. Pairs of biotinylated adaptor strands from the library are spotted as microarray and aligned to the PDMS chip. The real image shows 1 out of 640 unit cells of the microfluidic chip. Each unit cell is divided into two chambers, i.e., the DNA storage chamber and the assay chamber. Microfluidic membrane valves separate the unit cells from each other (V1) and the DNA storage and assay chambers (V2). In the first step, a layered pull-down assay is developed using the button valve on the epoxy-coated glass surface within all assay chambers of the chip (see main text). NeutrAvidin forms the reactive top layer of the pull-down assay. Next, biotinylated adaptors are resolved from the microarray spot in the DNA storage chamber and allowed to diffuse to the pull-down area in the assay chamber. Thus, an array containing all binary adaptor combinations is created. A pair of connectors to complement the adaptors was introduced from the outside of the chip to obtain fully assembled PLA templates. (B2) For the spPLA test system, pairs of connector strands from the library are spotted on the microarray and aligned to the PDMS chip. Similar to B1, a pull-down assay is developed on a NeutrAvidin-reactive surface. In subsequent washing steps, an antibody “sandwich” was developed to detect vascular endothelial growth factor (VEGF). The two anti-VEGF antibodies from different hosts were labeled with the adaptor strands. Combinations of adaptor strands within the antibody sandwich were achieved through fluidic multiplexing on the chip (see main text). Next, the connector pairs were resolved and allowed to diffuse to the pull-down area and fully assemble PLA templates. (C) After connector hybridization, fluids for PLA template ligation, amplification, and product detection are perfused in automated washing steps.

For the integration of a spRCA and spPLA, we used the microfluidic flow circuitry of a previously designed PDMS chip for testing binary biomolecular interactions [21]. The multilayer PDMS chip contained 640 parallel working unit cells, where each unit cell is divided into two chambers, i.e., a DNA storage chamber and an assay chamber (see Figure 3B). The combined volume of both chambers was 1.5 nL. Here, we used the available chip hardware and re-engineered the biochemical software. Figure 3 shows the integrated workflow for spRCA and spPLA. The workflow starts by spotting a DNA microarray of either pairs of adaptors or connectors on an epoxy-coated glass slide. Onto the microarray, the PDMS chip is aligned and heat-bonded (Figure 3A). Through alignment of the chip to the microarray, spots are located at the bottom of the DNA storage chambers. Before the DNA strands are spatially resolved in a buffered solution, a miniaturized pull-down assay is developed in the assay chamber of each unit cell.

To achieve this, sequential flush cycles were used to couple biotinylated BSA and NeutrAvidin to the epoxy-coated glass surface of the chip. In all assay chambers, a small circular area (Ø 75 µm2) of the top layer, i.e., reactive NeutrAvidin, was again passivated with biotinylated BSA. The circular surface area was protected during the passivation step through actuation of a pneumatic membrane valve, named button valve (see Figure 3B). Reactive NeutrAvidin was used as solid-phase anchor to assemble the different test systems.

### spRCA test system

To test the orthogonality of the *in silico* generated PLA templates, we randomly selected 10 out of the 24 generated PLA templates. The 20 adaptor strands of the 10 templates were synthesized with a biotin modification at their 5′ end. All 100 pairwise combinations (see Figure 1) were spotted in quadruplets onto a microarray using solutions containing equal concentrations of the two adaptors. In addition, each sub-array contained 20 spots with the single adaptors only, 20 spots with a short, unspecific biotinylated DNA strand, and 20 spots with no DNA. After bonding of the PDMS chip to the microarray the solid phase anchor NeutrAvidin was deposited within the assay chamber of each unit cell. In a next step the adaptor DNA strands were spatially dissolved from the spots by filling the DNA storage chambers with PBS solution. Through diffusion within one microfluidic unit cell, biotinylated adaptors are pulled down to the reactive NeutrAvidin surface. Because the concentration of the adaptors during the spot process was the same, we assumed that equal amounts of the two adaptors bound to the reactive NeutrAvidin surface (see Figure 3 B1). A pair of coding and detection connector was introduced to the chip and allowed to hybridize to the adaptor array. Next, a ligation and RCA reaction was performed in parallel within all unit cells of the microfluidic chip. Positive RCA events were detected with a fluorescence probe (see Figure 3C). Cross-contamination between unit cells during all flush steps was avoided through actuation and release of the button valves, which protected the pull-down area.

**Figure 4:**
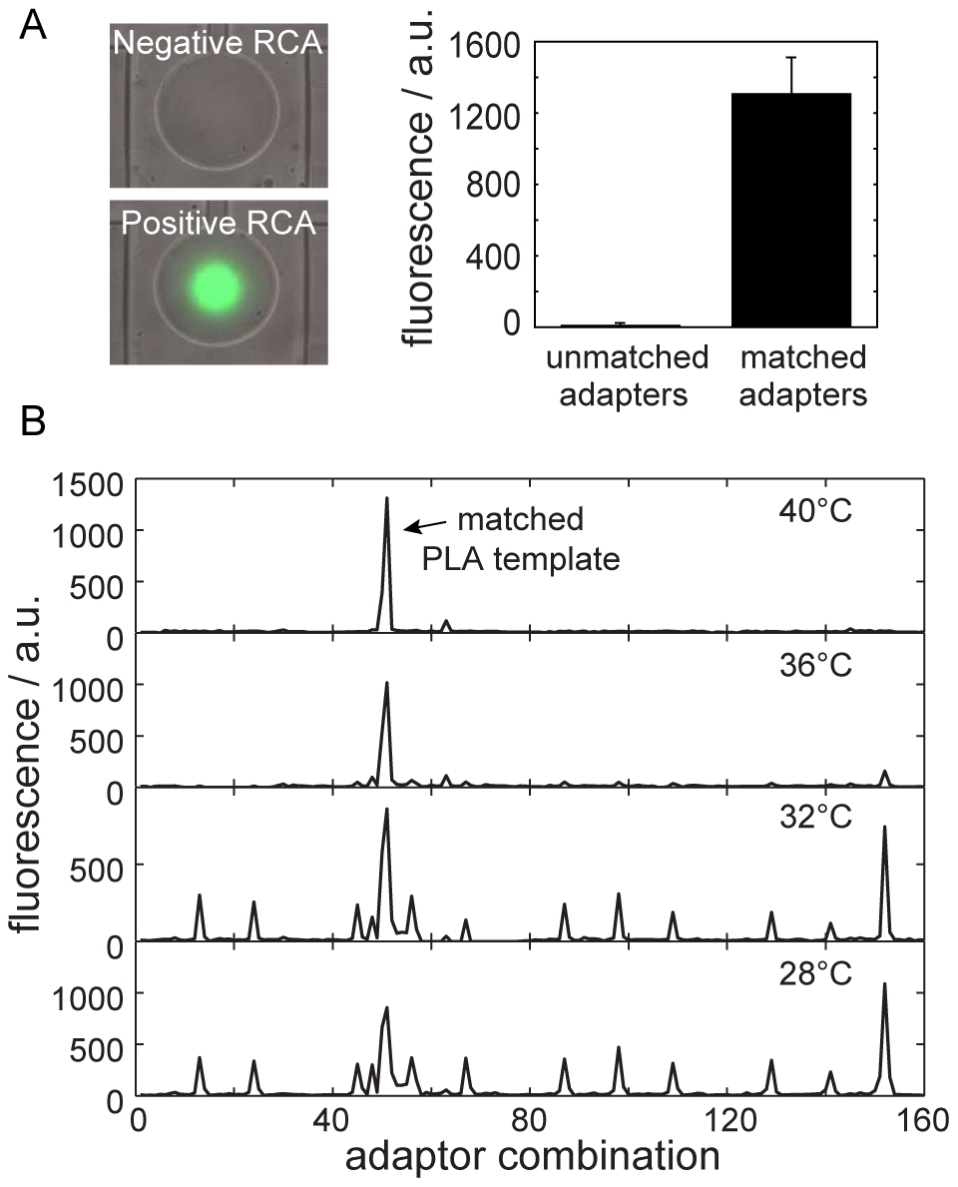
On-chip solid-phase rolling-circle amplification (spRCA) system for testing proximity ligation assay (PLA) templates. (A) The image shows a merged fluorescence and bright-field micrograph of two circular pull-down areas within representative unit cells on the chip. In the lower and upper images, positive and negative RCA events were obtained with matched and mismatched shared connectors (SCs), respectively. The bar graph shows background corrected fluorescence signal corresponding to the pull-down area of the left images. Error bars are calculated from four repeats. The scale bar represents 75 µm. (B) Temperature dependence of a PLA template assembling in a spRCA reaction. The graphs show the local background corrected fluorescence signals of 160 spRCA reactions, including the screening of one CC/DC pair, of 100 binary adaptor combinations from the selected 10 PLA templates. Furthermore, 20 single adaptors, 20 non-specific DNA strands, and 20 tests without DNA were screened against the same connector pair as control.

To test the specificity of the generated PLA templates we determined the temperature dependence of the spRCA reaction. Therefore one CC/DC pair was screened against the array of the 100 adaptor combinations on the chip at hybridization temperatures of 40°C, 36°C, 32°C, and 28°C. Figure 4B shows the corresponding fluorescence signals of the four spRCA experiments at different hybridization temperatures. Upon decreasing the hybridization temperature, the false-positive rate of the spRCA reaction strongly increased. We repeated this experiment with two further CCs and used the false positive events at 28°C for analysis of the AS sites. In 85% of the false positive cases, one adaptor exhibited a matching AS for the screened CC. Sequence analysis of the remaining false-positive events could not reveal a systematic distance parameter or a position dependence of minimal-matching nucleotides towards the 3′ and 5′ ends within AS sites.

In the following, we measured the reactivity of all 100 pairwise adaptor combinations against the 10 CCs within a spRCA reaction at 40°C. The screening was performed in triplicates for each combination, where replicates were located on different chip runs. Standard deviations between the three replicates were about 10% of the raw signals. Although, the chip-to-chip correlation was high with correlation coefficients over 0.98 (File S4) the absolute values of positive and negative spRCA could differ. The main reason for this is the variance of the adaptor concentration on the pull-down area due efficiency changes of the spotting and re-dissolving process of the adaptors. To compare chip experiments we normalized the fluorescence data by calculating a *Z*-score for each spRCA reaction, *Z* = (*I*−μ)/σ where *I*, *μ*, and σ is the background corrected fluorescence signal from a pull-down area, the mean value of all fluorescence signals from the chip run, and the standard deviation of the fluorescence signals from the chip run, respectively. Population difference between high and low *Z*-scores allows to distinguish between positive and negative spRCAs over different chip runs. The raw data is given in File S5.

**Figure 5:**
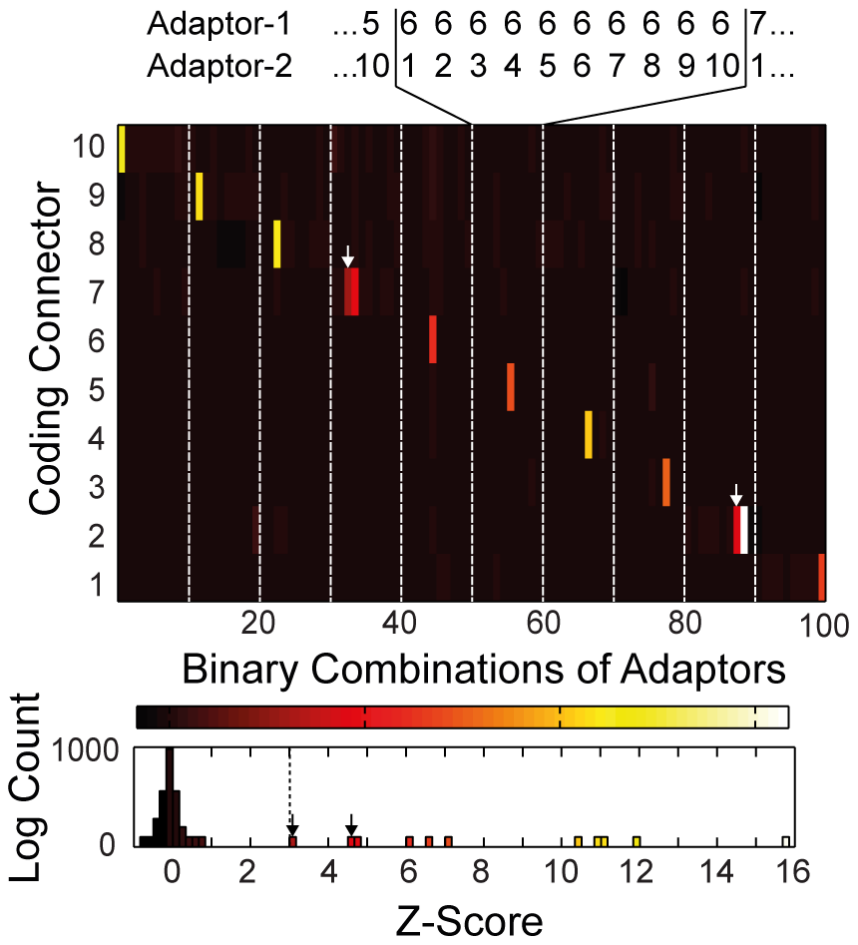
PLA template library screen with the solid-phase rolling-circle amplification (spRCA) system. The matrix shows the *Z*-scores for the RCA reactions of 100 adaptor pairs screened against 10 coding connectors (CCs) from 10 *in silico* generated PLA templates. The detection connector sequence was kept constant in all RCA reactions. Diagonal elements of the matrix (white bars) are the 10 matched adaptor/connector pairs. Red bars in the matrix indicate false-positive events from mismatched adaptor/SC combinations. The lower histogram presents the absolute values of the *Z*-score for the complete screening.

The *Z*-score results for the cross-reactivity screening of the PLA templates in a matrix format are shown in figure 5. The diagonal elements of the matrix are the *Z*-scores for the fluorescence signals of the matched PLA templates (true psoitives). Two spRCA reactions between mismatched adaptor/connector pairs had a *Z*-score higher than 3 (highlighted in red). We considered these adaptor/connector combinations as false-positives. Thus, 8 out of 10 designed PLA templates were orthogonal to each other at the given experimental conditions.

### spPLA test system

The microfluidic spRCA test system is a rapid and cost-effective method to identify non-orthogonal DNA elements in a multiplexing PLA template library; however, the assay is limited to DNA elements. To investigate the functionality, orthogonality, and efficiency of the eight orthogonal PLA templates from the spRCA in a PLA reaction, we developed a spPLA system on a microfluidic chip. To achieve this, combinations of the eight CC/DC pairs were spotted in repeats onto a microarray from solutions containing equal concentrations of the two connectors. Again, control spots with short, unspecific DNA strands and control spots with no DNA were included. After bonding of the PDMS chip to the microarray the same pull-down chemistry as for the spRCA was deposited within the unit cells. In difference to the spRCA a biotinylated anti-goat antibody was coupled to the solid phase anchor NeutrAvidin under the button area within all unit cells on chip.

In parallel, the 2×8 adaptors of the eight prescreened PLA templates were conjugated to polyclonal anti-VEGF antibody (Ab). The first adaptor set was conjugated to an anti-VEGF Ab from goat and the second adaptor set to an anti-VEGF Ab from rabbit (adaptor orientations are given in the SI). The 64 adaptor combinations of the two sets were tested in a sandwich antibody assay containing the two anti-VEGF Abs and a human recombinant VEGF. While combinations of adaptor pairs in the previous spRCA screen were established solely by the microarray spotting technique, we generated combinations of adaptor-labeled anti-VEGF Ab pairs by fluidic multiplexing. This was achieved by exploiting integrated PDMS membrane valves, which separated the chip into 8 equal sub-sections of unit cells.

In one chip run, one adaptor labeled anti-VEGF Ab from goat build the bottom of the antibody sandwich in all unit cells. In the following VEGF was introduced, where the concentration was chosen to saturate the binding sites of the deposited anti-VEGF Ab. Eight anti-VEGF antibodies from rabbit with the different labeled adaptors were then introduced to the sub-sections of the chip. After deposition of the VEGF antibody sandwiches, the connector strands were re-dissolved and allowed to diffuse from the DNA storage to the assay chamber within a microfluidic unit cell. Figure 3B2 summarizes the biochemical workflow. The amplification and detection of matched PLA templates was performed similar to the spRCA test system (Figure 3C). In total, eight chips were required to screen the combinatorial space of the PLA template library.

**Figure 6:**
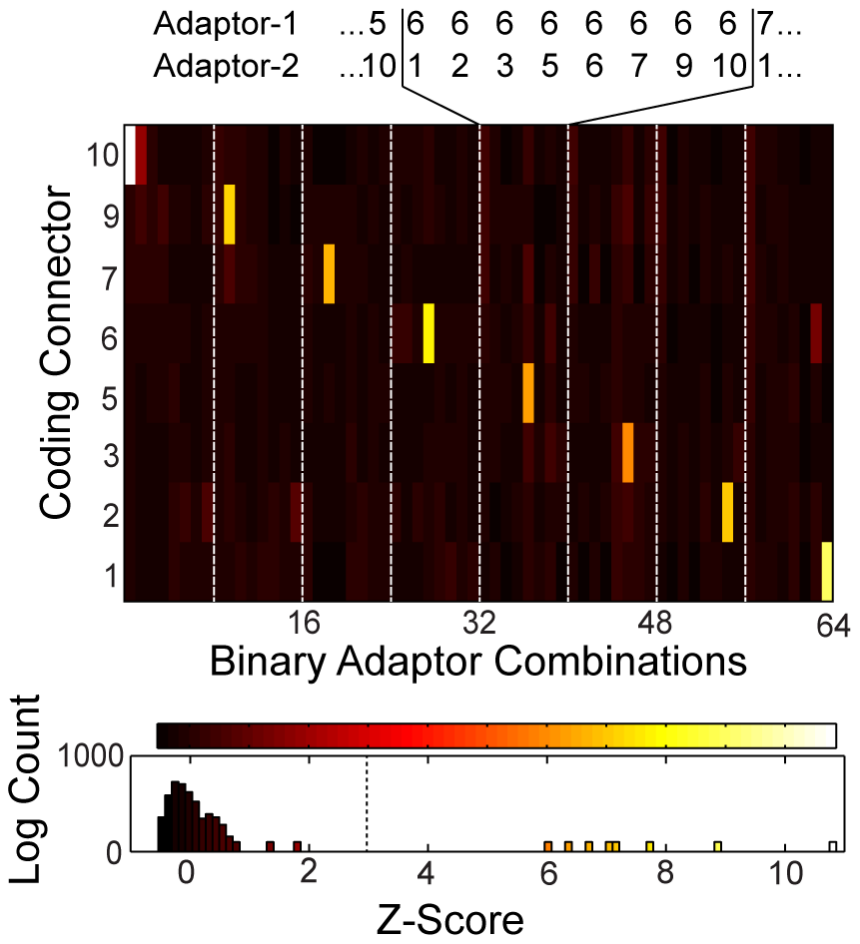
PLA template library screen with the solid phase proximity ligation assay (spPLA) system. The matrix shows the *Z*-scores for the PLA reactions of 64 antibody pairs with the same adaptors as in Figure 4 screened against eight coding connectors (CC) from the *in silico* generated PLA templates. The detection connector sequence was kept constant in all rolling-circle amplification (RCA) reactions. Diagonal elements of the matrix (white bars) are the eight matched adaptor/connector pairs. The numbering of the adaptor and connector is the same as the numbering of the PLA templates from Figure 4B. The plates 4 and 8 were removed in this screening because of false-positive reaction results in the RCA screening. The lower histogram presents the absolute values of the *Z*-scores for the complete screening.

The *Z*-score results for the cross-reactivity screening of the PLA templates in a matrix format in shown in figure 6. The diagonal elements of the matrix are the *Z*-scores for the fluorescence signals of the matched PLA templates (white bars) in a spPLA reaction. The lower histogram of Figure 6 shows the absolute *Z*-score results of the screening, which demonstrates a clear signal separation of the matched and unmatched PLA templates. No false-positive or -negative reactions were observed for the eight PLA templates when using a Z-score cut-off value of 3. The raw data is given in File S6.

## Discussion

Protein analytical technologies that offer high sensitivity and specificity at high throughput are in great demand for cellular network and diagnostic studies [26]. Here, we have developed a general design procedure to generate DNA multiplexing libraries for the PLA. To prove the orthogonality, functionality, and efficiency of PLA templates, we concomitantly engineered two solid-phase test systems on a microfluidic large–scale integration chip platform. The synergy between assays and the microfluidic technology was used to characterize the *in silico* PLA template design procedure on DNA and antibody levels.

While the orthogonality of an antibody library can only be determined experimentally, it is common to predict the orthogonality of DNA molecules in a composite with thermodynamic models [27]. Here, we showed that it is possible to obtain orthogonal AS sites by using a random generation process and a free energy minimization method for hybridization events between mismatched DNA sequences. The computational approach accounted for specific requirements of the PLA templates by including a filter function for DNA length, self-folding, and number of interacting DNA strands. It is close to assume that the ideal AS sites for assembling PLA templates in a multiplexing library exhibit the common properties: equal *T*_m_ values, high hybridization efficiency, and the largest possible sequence distance among each other. Centrally, our filter function did not include restrains for the *T*_m_ value or Hamming distance of AS sites. The Hamming distance is a sequence distance parameter counting the number of positions two equally long DNA strands differs in their sequence. The parameters were excluded since initial runs of the design procedure with restrained *T*_m_ and Hamming distance could not find solutions for PLA template libraries larger than 24. Consequently, the *T*_m_ values between the AS sites in the generated PLA template library deviated by ±3.5°C from the calculated mean of 34.5°C for an 11-base-pair-long AS site. The Hamming distance between all AS sites in the library varied between 3 and 11, and the longest contiguous match was 6 bases, which was 54% of an AS site.

The specificity of the DNA components of a PLA template depends on two sequential reactions: a hybridization reaction between four single DNA strands and a ligation reaction in order to form a circular DNA fragment. It has to be noted that the ligation of the two connectors leads to an increase of the *T*_m_ value between adaptor and connector strands and therewith the thermodynamic possibility to accept a base-pair mismatch. Furthermore, ligases accept base-pair mismatches or gaps at the 3′ hydroxyl end or nick sites [28]. For these reasons, designed multiplexing PLA template libraries will require experimental verification.

An important result of the experimental verification of the orthogonality of the PLA templates was a higher false-positive rate for adaptor/connector combinations with one matching AS site at annealing temperatures below the mean calculated *T*_m_ value of an 11-base-pair-long DNA strand. Competition between connector strands with one or even two AS sites will be often encountered in a multiplex approach. Importantly, only upon increasing the annealing temperature above the mean calculated *T*_m_ value of the AS sites, the false-positive rate of spRCA reactions was eliminated. We noted that higher annealing temperature resulted in an increase rather than a decrease in fluorescence signals of spRCA reactions with matched adaptor/connector pairs. The efficiency difference can either be explained by the possibility that the actual *T*_m_ values differ from the simulated values due the influence of the salt ions in the experiments or by the interworking hybridization and ligation reaction. At higher temperatures, only a small proportion of matched connectors hybridizes to the adaptors; however, upon ligation, the melting temperature of the connectors is shifted and, consequently, the hybridization equilibrium shifts towards correctly assembled PLA templates. To test further the competition between connector strands with one and two matching AS sites within a PLA template library, we mixed all eight orthogonal CCs with the constant DC and screened the composite against the binary adaptor library on the chip. The resulting fluorescence signals for all eight matched spRCAs exhibited the same intensity (∼5%), as measured in the absence of the competing SCs at 40°C (see SI).

In a two-step process, we brought evidence for the functionality, orthogonality, and efficiency of the designed PLA templates for multiplexing. In the first step, a false- and true-positive rate of 0.2% and 100% was determined, respectively for the PLA template design procedure with the spRCA test system on chip. In the second step, we confirmed the found orthogonal PLA templates in a full spPLA reaction. Orthogonal PLA templates within spRCA were also orthogonal in spPLA, which allows the conclusion that it is sufficient to evaluate the DNA elements of a PLA template library on a DNA level. This has the advantage of testing DNA elements of the PLA without coupling to antibodies, which reduces the time and cost for establishing larger PLA template libraries applicable for higher-throughput science.

In summary, we conclude that the experimental verification of the orthogonality of PLA templates proved the *in silico* free energy minimization procedure of cross-hybridization events. Our antibody target encoding strategy is suitable for multiplexing the PLA with independent and shared PLA target libraries. Miniaturization of the spPLA on a microfluidic chip created a synergy between a protein assay technology and an automation technology. It has been shown that it is of importance to control the environmental conditions of the complex PLA workflow in order to obtain high-quality data from multiplexed PLAs. The presented microfluidic platform enables the test of PLA template designs with DNA strands of different length, number of oligonucleotide strands, or encoding sequences. More importantly, the developed integrated surface chemistry for the spPLA on a microfluidic chip can be applied to high-throughput analytical protein detection methods on new mLSI chips for cell lysates and other fluids on microfluidic chip platforms.

## Supporting Information

Table S1 DNA sequences of the generated PLA templates. The numberring of the adaptor and conncetors matching the numbers used in figure 5 and 6 of the main text.
(PDF)

File S1 Source code of the program to generate PLA templates following the approach given in figure 2. Help and annotation notes are given in the file.
(ZIP)

File S2 Microfluidic workflow of the solid phase RCA with a chemical reagent list.
(PDF)

File S3 Microfluidic workflow of the solid phase PLA with a chemical reagent list.
(PDF)

File S4 Chip-to-chip correlation for the spRCA reactions. The graph shows the correlation of the spRCA fluorescence signals between repeats for the complete spRCA library screen. Repeats were performed within different chip runs.
(PDF)

File S5 Raw data file containing the fluorescence signals of the spRCA reactions for the complet DNA template library screen.
(TXT)

File S6 Raw data file containing the fluorescence signals of the spPLA reactions for the selected DNA template library screen.
(TXT)

## References

[1] Juncker D, Bergeron S, Laforte V, Li H (2014) ScienceDirectCross-reactivity in antibody microarrays and multiplexed sandwich assays: shedding light on the dark side of multiplexing. Current Opinion in Chemical Biology 18: 29–37 10.1016/j.cbpa.2013.11.012 24534750

[2] Towbin H, Staehelin T, Gordon J (1979) Electrophoretic transfer of proteins from polyacrylamide gels to nitrocellulose sheets: procedure and some applications. Proc Natl Acad Sci USA 76: 4350–4354. 388439 10.1073/pnas.76.9.4350 PMC411572

[3] Yalow RS, Berson SA (1960) Immunoassay of Endogenous Plasma Insulin in Man. J Clin Invest 39: 1157–1175 10.1172/JCI104130 13846364 PMC441860

[4] Aebersold R, Mann M (2003) Mass spectrometry-based proteomics. Nature 422: 198–207 10.1038/nature01511 12634793

[5] Meier M, Sit RV, Quake SR (2013) Proteome-wide protein interaction measurements of bacterial proteins of unknown function. PNAS 110: 477–482 10.1073/pnas.1210634110/-/DCSupplemental/pnas.201210634SI.pdf 23267104 PMC3545810

[6] Han KN, Li CA, Seong GH (2013) Microfluidic Chips for Immunoassays. Annual Review of Analytical Chemistry 6: 119–141 10.1146/annurev-anchem-062012-092616 23495732

[7] Yu H, Braun P, Yildirim MA, Lemmens I, Venkatesan K, et al (2008) High-quality binary protein interaction map of the yeast interactome network. Science 322: 104–110 10.1126/science.1158684 18719252 PMC2746753

[8] Fredriksson S, Gullberg M, Jarvius J, Olsson C, Pietras K, et al (2002) Protein detection using proximity-dependent DNA ligation assays. Nat Biotechnol 20: 473–477 10.1038/nbt0502-473 11981560

[9] Fire A, Xu SQ (1995) Rolling replication of short DNA circles. Proc Natl Acad Sci USA 92: 4641–4645. 7753856 10.1073/pnas.92.10.4641 PMC42000

[10] Gustafsdottir SM (2006) Detection of Individual Microbial Pathogens by Proximity Ligation. Clinical Chemistry 52: 1152–1160 10.1373/clinchem.2005.065847 16723682

[11] Ericsson O, Jarvius J, Schallmeiner E, Howell M, Nong RY, et al (2008) A dual-tag microarray platform for high-performance nucleic acid and protein analyses. Nucleic Acids Res 36: e45–e45 10.1093/nar/gkn106 18346972 PMC2377440

[12] Söderberg O, Gullberg M, Jarvius M, Ridderstråle K, Leuchowius K-J, et al (2006) Direct observation of individual endogenous protein complexes in situ by proximity ligation. Nat Meth 3: 995–1000 10.1038/nmeth947 17072308

[13] Söderberg O, Leuchowius K-J, Gullberg M, Jarvius M, Weibrecht I, et al (2008) Characterizing proteins and their interactions in cells and tissues using the in situ proximity ligation assay. Methods 45: 227–232 10.1016/j.ymeth.2008.06.014 18620061

[14] Darmanis S, Nong RY, Vänelid J, Siegbahn A, Ericsson O, et al (2011) ProteinSeq: High-Performance Proteomic Analyses by Proximity Ligation and Next Generation Sequencing. PLoS ONE 6: e25583 10.1371/journal.pone.0025583 21980495 PMC3183061

[15] Nong RY, Wu D, Yan J, Hammond M, Gu GJ, et al (2013) Solid-phase proximity ligation assays for individual or parallel protein analyses with readout via real-time PCR or sequencing. Nat Protoc 8: 1234–1248 10.1038/nprot.2013.070 23722261

[16] Lundberg M, Thorsen SB, Assarsson E, Villablanca A, Tran B, et al (2011) Multiplexed Homogeneous Proximity Ligation Assays for High-throughput Protein Biomarker Research in Serological Material. Molecular & Cellular Proteomics 10: M110.004978–M110.004978 10.1074/mcp.M110.004978 PMC3069344 21242282

[17] Leuchowius K (2013) Molecular & Cellular Proteomics 2013. Leuchowiu 1–25.

[18] Blazek M, Betz C, Hall MN, Reth M, Zengerle R, et al (2013) Proximity Ligation Assay for High-content Profiling of Cell Signaling Pathways on a Microfluidic Chip. Mol Cell Proteomics 12: 3898–3907 10.1074/mcp.M113.032821 24072685 PMC3861732

[19] Schweitzer B, Roberts S, Grimwade B, Shao W (2002) Multiplexed protein profiling on microarrays by rolling-circle amplification. Nature 10.1016/S0958-1669(02)00019-8 PMC2858761 11923841

[20] Fredriksson S, Dixon W, Ji H, Koong AC, Mindrinos M, et al (2007) Multiplexed protein detection by proximity ligation for cancer biomarker validation. Nat Meth 10.1038/nmeth1020 17369836

[21] Maerkl SJ, Quake SR (2007) A Systems Approach to Measuring the Binding Energy Landscapes of Transcription Factors. Science 315: 233–237 10.1126/science.1131007 17218526

[22] Lorenz R, Bernhart S (2011) ViennaRNA Package 2.0. … for Molecular Biology 10.1186/1748-7188-6-26 PMC3319429 22115189

[23] Unger MA, Chou HP, Thorsen T, Scherer A, Quake SR (2000) Monolithic microfabricated valves and pumps by multilayer soft lithography. Science 288: 113–116. 10753110 10.1126/science.288.5463.113

[24] Tafer H, Hofacker IL (2008) RNAplex: a fast tool for RNA-RNA interaction search. Bioinformatics 24: 2657–2663 10.1093/bioinformatics/btn193 18434344

[25] Zhang DY, Chen SX, Yin P (2012) Optimizing the specificity of nucleic acid hybridization:. 1–7 10.1038/nchem.1246 PMC4238961 22354435

[26] Whitesides GM (2006) The origins and the future of microfluidics. Nature 442: 368–373 10.1038/nature05058 16871203

[27] Dirks RM, Bois JS, Schaeffer JM, Winfree E, Pierce NA (2007) Thermodynamic Analysis of Interacting Nucleic Acid Strands. SIAM Rev 49: 65–88 10.1137/060651100

[28] Kim J, Mrksich M (2009) Profiling the selectivity of DNA ligases in an array format with mass spectrometry. Nucleic Acids Res 38: e2–e2 10.1093/nar/gkp827 19854942 PMC2800213

