# Supplementary material for: Design and Validation of DNA Libraries for Multiplexing Proximity Ligation Assays
Source: PLoS One. 2014 Nov 11;9(11):e112629. doi: 10.1371/journal.pone.0112629 (PMC4227721; doi:10.1371/journal.pone.0112629)
Supplement: Table S1 — DNA sequences of the generated PLA templates. The numberring of the adaptor and conncetors matching the numbers used in figure 5 and 6 of the main text. (PDF) [file pone.0112629.s001.pdf]

	Sequence	DNA Assay		Antibody Assay		
		5'	3'	5'	3'	Conjugated to
Fluorescent probe	CAGTGAATGCGAGTCCGTCT	FAM	-			
Long Connector	CCCAGAGTTTTCAGTGAATGCGAGTCCGTCTAAGAG					
	AGTAGTACAGCAGCCGTCCGTCCAAATGA	[Phos]	-	[Phos]	-	
Adaptor-1 1	AAAAAAAAAAAAAACTCTGGGGTATGCTATGC	[BtnTg]	-	[ThiC6]	-	Goat anti-VEGF121
Adaptor-2 1	AAAAAAAAAATACCCGATTGATCATTTGGACG	[BtnTg]	-	[ThiC6]	-	Rabbit anti-VEGF165
Short connector 1	TCAATCGGGTANNNGCATAGCATAC	[Phos]	-	[Phos]	-	
Adaptor-1 2	AAAAAAAAAAAAAACTCTGGGCTGACATACTG	[BtnTg]	-	[ThiC6]	-	Goat anti-VEGF121
Adaptor-2 2	AAAAAAAAAATCCTAGCGATTTCATTTGGACG	[BtnTg]	-	[ThiC6]	-	Rabbit anti-VEGF165
Short connector 2	AATCGCTAGGANNNCAGTATGTCAG	[Phos]	-	[Phos]	-	
Adaptor-1 3	AAAAAAAAAAAAAACTCTGGGCCGAATAGGTG	[BtnTg]	-	[ThiC6]	-	Goat anti-VEGF121
Adaptor-2 3	AAAAAAAAAACTTTGAAGCACTCATTTGGACG	[BtnTg]	-	[ThiC6]	-	Rabbit anti-VEGF165
Short connector 3	GTGCTTCAAAGNNNCACCTATTCGG	[Phos]	-	[Phos]	-	
Adaptor-1 4	AAAAAAAAAAAAAACTCTGGGGTAACGTTCGT	[BtnTg]	-			not used
Adaptor-2 4	AAAAAAAAAACTTGACAGAACTCATTTGGACG	[BtnTg]	-			not used
Short connector 4	GTTCTGTCAAGNNNACGAACGTTAC	[Phos]	-			not used
Adaptor-1 5	AAAAAAAAAAAAAACTCTGGGTGGTTCAACTG	[BtnTg]	-	[ThiC6]	-	Goat anti-VEGF121
Adaptor-2 5	AAAAAAAAAAGTAATGACACCTCATTTGGACG	[BtnTg]	-	[ThiC6]	-	Rabbit anti-VEGF165
Short connector 5	GGTGTCATTACNNNCAGTTGAACCA	[Phos]	-	[Phos]	-	
Adaptor-1 6	AAAAAAAAAAAAAACTCTGGGCTATTGCATGG	[BtnTg]	-	[ThiC6]	-	Goat anti-VEGF121
Adaptor-2 6	AAAAAAAAAAGCGAAATACCTTCATTTGGACG	[BtnTg]	-	[ThiC6]	-	Rabbit anti-VEGF165
Short connector 6	AGGTATTTCGCNNNCCATGCAATAG	[Phos]	-	[Phos]	-	
Adaptor-1 7	AAAAAAAAAAAAAACTCTGGGGAGACTTACGA	[BtnTg]	-	[ThiC6]	-	Goat anti-VEGF121
Adaptor-2 7	AAAAAAAAAAGCGATAATCAGTCATTTGGACG	[BtnTg]	-	[ThiC6]	-	Rabbit anti-VEGF165
Short connector 7	CTGATTATCGCNNNTCGTAAGTCTC	[Phos]	-	[Phos]	-	
Adaptor-1 8	AAAAAAAAAAAAAACTCTGGGCTATGAAACGC	[BtnTg]	-			not used
Adaptor-2 8	AAAAAAAAAACTCTATACGTGTCATTTGGACG	[BtnTg]	-			not used
Short connector 8	CACGTATAGAGNNNGCGTTTCATAG	[Phos]	-			not used
Adaptor-1 9	AAAAAAAAAAAAAACTCTGGGGCCCTTGTTAA	[BtnTg]	-	[ThiC6]	-	Goat anti-VEGF121
Adaptor-2 9	AAAAAAAAAAGACCATTTGTGTCATTTGGACG	[BtnTg]	-	[ThiC6]	-	Rabbit anti-VEGF165
Short connector 9	CACAAATGGTCNNNTTAACAAGGGC	[Phos]	-	[Phos]	-	
Adaptor-1 10	AAAAAAAAAAAAAACTCTGGGCAGGAATATGC	[BtnTg]	-	[ThiC6]	-	Goat anti-VEGF121
Adaptor-2 10	AAAAAAAAAATTTACCGTGGTCATTTGGACG	[BtnTg]	-	[ThiC6]	-	Rabbit anti-VEGF165
Short connector 10	CCACGGTAAATNNNGCATATTCCTG	[Phos]	-	[Phos]	-	
