# Supplementary material for: Design and Validation of DNA Libraries for Multiplexing Proximity Ligation Assays
Source: PLoS One. 2014 Nov 11;9(11):e112629. doi: 10.1371/journal.pone.0112629 (PMC4227721; doi:10.1371/journal.pone.0112629)
Supplement: File S1 — Source code of the program to generate PLA templates following the approach given in figure 2. Help and annotation notes are given in the file. (ZIP) [file pone.0112629.s002.zip › generate_PLA_lib/doc/html/DNA__manipulation_8h_source.html]

generate\_PLA\_lib: include/DNA\_manipulation.h Source File


|  |
| --- |
| generate\_PLA\_lib  Generation of a library of DNA sequences suitable for multiplexing PLA |


- Main Page
- Files

- File List
- File Members

All Files Functions Variables Macros Pages

- include

DNA\_manipulation.h

1 #ifndef DNA\_MANIP

2 #define DNA\_MANIP

3

4 void mutate(char \*, int);

5 void parse\_DNA(char \*);

6 char \* reverse\_complement(char \*);

7 float get\_GC(char \*);

8 float RNAplex(char\*, char\*);

9 #endif

reverse\_complement

char \* reverse\_complement(char \*DNA)

Computes thes reverse complement of a DNA sequence.

**Definition:** DNA\_manipulation.c:92

RNAplex

float RNAplex(char \*seq1, char \*seq2)

Function to get RNAplex score of two sequences.

**Definition:** DNA\_manipulation.c:157

parse\_DNA

void parse\_DNA(char \*DNA)

parse a DNA sequence, removes non-ACTG chars and capitalizes.

**Definition:** DNA\_manipulation.c:34

get\_GC

float get\_GC(char \*DNA)

Computes the GC-content of a DNA sequence.

**Definition:** DNA\_manipulation.c:128

mutate

void mutate(char \*DNA, int count)

mutate a DNA sequence

**Definition:** DNA\_manipulation.c:21


---

Generated on Mon May 12 2014 15:06:53 for generate\_PLA\_lib by  

 1.8.6
