# Supplementary material for: Design and Validation of DNA Libraries for Multiplexing Proximity Ligation Assays
Source: PLoS One. 2014 Nov 11;9(11):e112629. doi: 10.1371/journal.pone.0112629 (PMC4227721; doi:10.1371/journal.pone.0112629)
Supplement: File S1 — Source code of the program to generate PLA templates following the approach given in figure 2. Help and annotation notes are given in the file. (ZIP) [file pone.0112629.s002.zip › generate_PLA_lib/doc/html/SeqCplxCst_8h_source.html]

generate\_PLA\_lib: include/SeqCplxCst.h Source File


|  |
| --- |
| generate\_PLA\_lib  Generation of a library of DNA sequences suitable for multiplexing PLA |


- Main Page
- Files

- File List
- File Members

All Files Functions Macros

- include

SeqCplxCst.h

1 #ifndef CONST

2 #define CONST

3

4 #define GC 50

5 #define GC\_VAR 6

6

7 #define NT 0.25

8 #define NT\_VAR 0.17

9

10 #endif


---

Generated on Tue May 6 2014 12:47:16 for generate\_PLA\_lib by  

 1.8.6
