# Supplementary material for: Design and Validation of DNA Libraries for Multiplexing Proximity Ligation Assays
Source: PLoS One. 2014 Nov 11;9(11):e112629. doi: 10.1371/journal.pone.0112629 (PMC4227721; doi:10.1371/journal.pone.0112629)
Supplement: File S1 — Source code of the program to generate PLA templates following the approach given in figure 2. Help and annotation notes are given in the file. (ZIP) [file pone.0112629.s002.zip › generate_PLA_lib/doc/html/files.html]

generate\_PLA\_lib: File List


|  |
| --- |
| generate\_PLA\_lib  Generation of a library of DNA sequences suitable for multiplexing PLA |


- Main Page
- Files

- File List
- File Members


All Files Functions Variables Macros Pages

File List

Here is a list of all documented files with brief descriptions:

[detail level 12]

|  |  |
| --- | --- |
| include |  |
| Check\_functions.c | This file contains the functions used by the algorithm to test annealiing sequence candidates |
| **Check\_functions.h** |  |
| DNA\_manipulation.c | Function to manipulate DNA sequences |
| **DNA\_manipulation.h** |  |
| main.c | Main algorithm file |


---

Generated on Mon May 12 2014 15:06:53 for generate\_PLA\_lib by  

 1.8.6
