# Supplementary material for: Design and Validation of DNA Libraries for Multiplexing Proximity Ligation Assays
Source: PLoS One. 2014 Nov 11;9(11):e112629. doi: 10.1371/journal.pone.0112629 (PMC4227721; doi:10.1371/journal.pone.0112629)
Supplement: File S1 — Source code of the program to generate PLA templates following the approach given in figure 2. Help and annotation notes are given in the file. (ZIP) [file pone.0112629.s002.zip › generate_PLA_lib/doc/html/main_8c.html]

generate\_PLA\_lib: main.c File Reference


|  |
| --- |
| generate\_PLA\_lib  Generation of a library of DNA sequences suitable for multiplexing PLA |


- Main Page
- Files

- File List
- File Members

All Files Functions Variables Macros Pages

Macros |
Functions |
Variables

main.c File Reference

Main algorithm file.
More...

`#include <stdlib.h>`  
`#include <stdio.h>`  
`#include <math.h>`  
`#include <string.h>`  
`#include <time.h>`  
`#include <omp.h>`  
`#include <signal.h>`  
`#include <sys/stat.h>`  
`#include <unistd.h>`  
`#include "include/DNA_manipulation.h"`  
`#include "include/Check_functions.h"`  
`#include "fold.h"`  
`#include "utils.h"`

|  |  |
| --- | --- |
| Macros | |
| #define | DC\_COUNT   1 |
|  | How many Detection Connector are considered in the run. |
|  | |
| #define | DC\_PROBE   20 |
|  | Length of probe sequence in nucleotides on Detection Connector. |
|  | |
| #define | DC\_SPACER   23 |
|  | Length of spacer after probe sequence on Detection Connector. |
|  | |
| #define | AN\_DC   0.30 |
|  | Maximum percentage of AS annealing to the constant region of Detection Connector. |
|  | |
| #define | SKIP\_MUTATION   10 |
|  | probability of skiping mutation of a sequence. Between 0 and 100 |
|  | |

|  |  |
| --- | --- |
| Functions | |
| void | sigint\_handler (int sig) |
|  | Function to catck kill signal (catching ctrl-c keys) and terminate the program cleanly without loosing the run. |
|  | |
| int | main (int argc, char \*argv[]) |
|  | Main function. More... |
|  | |

|  |  |
| --- | --- |
| Variables | |
| int | exit\_print =0 |
|  | Status variable to exit without loosing the run. |
|  | |

## Detailed Description

Main algorithm file.

Author
:   Nicolas Gobet

Version
:   1.0

Date
:   06 may 2014 Publication: N. Gobet et al. Analytical Chemistry 2014

The file consists of two main loops. The first for initializing a set of PLA fragments fulfilling user-defined conditions. The second loop maximize the gap between RNAplex scores of expected cross hybridization between mismatched DNA strands. All ssDNA specific scores are computed using ViennaRNA and MFE values.

## Function Documentation

|  |  |  |  |
| --- | --- | --- | --- |
| int main | ( | int | *argc*, |
|  |  | char \* | *argv*[] |
|  | ) |  |  |

Main function.

Parameters
:   |  |  |
    | --- | --- |
    | argc | How many parameters entered by user |
    | argv | Parameters entered by user |


---

Generated on Mon May 12 2014 15:06:53 for generate\_PLA\_lib by  

 1.8.6
