# Supplementary material for: Design and Validation of DNA Libraries for Multiplexing Proximity Ligation Assays
Source: PLoS One. 2014 Nov 11;9(11):e112629. doi: 10.1371/journal.pone.0112629 (PMC4227721; doi:10.1371/journal.pone.0112629)
Supplement: File S1 — Source code of the program to generate PLA templates following the approach given in figure 2. Help and annotation notes are given in the file. (ZIP) [file pone.0112629.s002.zip › generate_PLA_lib/doc/html/globals_func.html]

generate\_PLA\_lib: File Members


|  |
| --- |
| generate\_PLA\_lib  Generation of a library of DNA sequences suitable for multiplexing PLA |


- Main Page
- Files

- File List
- File Members

- All
- Functions
- Variables
- Macros


All Files Functions Variables Macros Pages

- ck\_cell\_score()
  : Check\_functions.c
- ck\_GC()
  : Check\_functions.c
- ck\_hairpin()
  : Check\_functions.c
- ck\_nt\_bias()
  : Check\_functions.c
- ck\_sec\_struct()
  : Check\_functions.c
- get\_GC()
  : DNA\_manipulation.c
- main()
  : main.c
- mutate()
  : DNA\_manipulation.c
- parse\_DNA()
  : DNA\_manipulation.c
- reverse\_complement()
  : DNA\_manipulation.c
- RNAplex()
  : DNA\_manipulation.c
- sigint\_handler()
  : main.c


---

Generated on Mon May 12 2014 15:06:54 for generate\_PLA\_lib by  

 1.8.6
