# Supplementary material for: Design and Validation of DNA Libraries for Multiplexing Proximity Ligation Assays
Source: PLoS One. 2014 Nov 11;9(11):e112629. doi: 10.1371/journal.pone.0112629 (PMC4227721; doi:10.1371/journal.pone.0112629)
Supplement: File S1 — Source code of the program to generate PLA templates following the approach given in figure 2. Help and annotation notes are given in the file. (ZIP) [file pone.0112629.s002.zip › generate_PLA_lib/doc/html/DNA__manipulation_8c.html]

generate\_PLA\_lib: include/DNA\_manipulation.c File Reference


|  |
| --- |
| generate\_PLA\_lib  Generation of a library of DNA sequences suitable for multiplexing PLA |


- Main Page
- Files

- File List
- File Members

All Files Functions Variables Macros Pages

- include

Functions

DNA\_manipulation.c File Reference

Function to manipulate DNA sequences.
More...

`#include <stdlib.h>`  
`#include <stdio.h>`  
`#include <string.h>`  
`#include <time.h>`  
`#include "utils.h"`

|  |  |
| --- | --- |
| Functions | |
| void | mutate (char \*DNA, int count) |
|  | mutate a DNA sequence More... |
|  | |
| void | parse\_DNA (char \*DNA) |
|  | parse a DNA sequence, removes non-ACTG chars and capitalizes. More... |
|  | |
| char \* | reverse\_complement (char \*DNA) |
|  | Computes thes reverse complement of a DNA sequence. More... |
|  | |
| float | get\_GC (char \*DNA) |
|  | Computes the GC-content of a DNA sequence. More... |
|  | |
| float | RNAplex (char \*seq1, char \*seq2) |
|  | Function to get RNAplex score of two sequences. More... |
|  | |

## Detailed Description

Function to manipulate DNA sequences.

Author
:   Nicolas Gobet

Version
:   0.9

Date
:   06 may 2014

## Function Documentation

|  |  |  |  |  |  |
| --- | --- | --- | --- | --- | --- |
| float get\_GC | ( | char \* | *DNA* | ) |  |

Computes the GC-content of a DNA sequence.

Parameters
:   |  |  |
    | --- | --- |
    | DNA | Pointer to the sequence |

Returns
:   GC-content, float

|  |  |  |  |
| --- | --- | --- | --- |
| void mutate | ( | char \* | *DNA*, |
|  |  | int | *count* |
|  | ) |  |  |

mutate a DNA sequence

Parameters
:   |  |  |
    | --- | --- |
    | DNA | Pointer to the sequence |
    | count | Not implemented |

|  |  |  |  |  |  |
| --- | --- | --- | --- | --- | --- |
| void parse\_DNA | ( | char \* | *DNA* | ) |  |

parse a DNA sequence, removes non-ACTG chars and capitalizes.

Parameters
:   |  |  |
    | --- | --- |
    | DNA | Pointer to the sequence |

|  |  |  |  |  |  |
| --- | --- | --- | --- | --- | --- |
| char\* reverse\_complement | ( | char \* | *DNA* | ) |  |

Computes thes reverse complement of a DNA sequence.

Parameters
:   |  |  |
    | --- | --- |
    | DNA | Pointer to the sequence |

Returns
:   pointer to the reverse complement sequence

|  |  |  |  |
| --- | --- | --- | --- |
| float RNAplex | ( | char \* | *seq1*, |
|  |  | char \* | *seq2* |
|  | ) |  |  |

Function to get RNAplex score of two sequences.

Parameters
:   |  |  |
    | --- | --- |
    | seq1 | pointer to the first sequence |
    | seq2 | pointer to the second sequence |

Returns
:   return score

This function is calling RNAplex function from ViennaRNA from the shell terminal and parsing result to extract score.


---

Generated on Mon May 12 2014 15:06:53 for generate\_PLA\_lib by  

 1.8.6
