# Supplementary material for: Design and Validation of DNA Libraries for Multiplexing Proximity Ligation Assays
Source: PLoS One. 2014 Nov 11;9(11):e112629. doi: 10.1371/journal.pone.0112629 (PMC4227721; doi:10.1371/journal.pone.0112629)
Supplement: File S1 — Source code of the program to generate PLA templates following the approach given in figure 2. Help and annotation notes are given in the file. (ZIP) [file pone.0112629.s002.zip › generate_PLA_lib/doc/html/Check__functions_8h_source.html]

generate\_PLA\_lib: include/Check\_functions.h Source File


|  |
| --- |
| generate\_PLA\_lib  Generation of a library of DNA sequences suitable for multiplexing PLA |


- Main Page
- Files

- File List
- File Members

All Files Functions Variables Macros Pages

- include

Check\_functions.h

1 #ifndef CK\_FCT

2 #define CK\_FCT

3

4 int ck\_hairpin(char \*);

5 int ck\_sec\_struct(char \*, int );

6 int ck\_cell\_score(int, int, int, int);

7 int ck\_GC(char \*);

8 int ck\_nt\_bias(char \*);

9 int ck\_RC(char \*, int);

10 #endif

ck\_nt\_bias

int ck\_nt\_bias(char \*DNA)

Function to check if single nucleotide bias is within a range.

**Definition:** Check\_functions.c:165

ck\_GC

int ck\_GC(char \*DNA)

Function to check if GC-content is within a range.

**Definition:** Check\_functions.c:152

ck\_hairpin

int ck\_hairpin(char \*cur\_struct)

Function to check if a DNA strand has hairpin.

**Definition:** Check\_functions.c:107

ck\_cell\_score

int ck\_cell\_score(int mut1, int mut2, int cur\_l, int cur\_c)

Function to determine id a cell of the score array has to be evaluated after mutating two sequences...

**Definition:** Check\_functions.c:134

ck\_sec\_struct

int ck\_sec\_struct(char \*cur\_struct, int where)

Function to check if a DNA strand has free ends.

**Definition:** Check\_functions.c:51


---

Generated on Mon May 12 2014 15:06:53 for generate\_PLA\_lib by  

 1.8.6
