# Supplementary material for: Design and Validation of DNA Libraries for Multiplexing Proximity Ligation Assays
Source: PLoS One. 2014 Nov 11;9(11):e112629. doi: 10.1371/journal.pone.0112629 (PMC4227721; doi:10.1371/journal.pone.0112629)
Supplement: File S1 — Source code of the program to generate PLA templates following the approach given in figure 2. Help and annotation notes are given in the file. (ZIP) [file pone.0112629.s002.zip › generate_PLA_lib/doc/html/Check__functions_8c.html]

generate\_PLA\_lib: include/Check\_functions.c File Reference


|  |
| --- |
| generate\_PLA\_lib  Generation of a library of DNA sequences suitable for multiplexing PLA |


- Main Page
- Files

- File List
- File Members

All Files Functions Variables Macros Pages

- include

Macros |
Functions

Check\_functions.c File Reference

This file contains the functions used by the algorithm to test annealiing sequence candidates.
More...

`#include <string.h>`  
`#include "DNA_manipulation.h"`

|  |  |
| --- | --- |
| Macros | |
| #define | FREE\_NT   13 |
|  | How many nucleotides have to be free on end of DNA fragments. |
|  | |
| #define | MAX\_HAIRPIN   0.1 |
|  | Maximum percentage of nucleotides folded when checking for hairpin. |
|  | |
| #define | GC   50 |
|  | Middle value for GC-content. |
|  | |
| #define | GC\_VAR   5 |
|  | GC-content of candidates can vary of +/- GC\_VAR from GC. |
|  | |
| #define | NT   0.25 |
|  | Middle value for single nucleotide content. |
|  | |
| #define | NT\_VAR   0.12 |
|  | single nucleotide content of candidates can vary of +/- NT\_VAR from NT |
|  | |

|  |  |
| --- | --- |
| Functions | |
| int | ck\_sec\_struct (char \*cur\_struct, int where) |
|  | Function to check if a DNA strand has free ends. More... |
|  | |
| int | ck\_hairpin (char \*cur\_struct) |
|  | Function to check if a DNA strand has hairpin. More... |
|  | |
| int | ck\_cell\_score (int mut1, int mut2, int cur\_l, int cur\_c) |
|  | Function to determine id a cell of the score array has to be evaluated after mutating two sequences. More... |
|  | |
| int | ck\_GC (char \*DNA) |
|  | Function to check if GC-content is within a range. More... |
|  | |
| int | ck\_nt\_bias (char \*DNA) |
|  | Function to check if single nucleotide bias is within a range. More... |
|  | |

## Detailed Description

This file contains the functions used by the algorithm to test annealiing sequence candidates.

Author
:   Nicolas Gobet

Version
:   1.0

Date
:   06 may 2014

## Function Documentation

|  |  |  |  |
| --- | --- | --- | --- |
| int ck\_cell\_score | ( | int | *mut1*, |
|  |  | int | *mut2*, |
|  |  | int | *cur\_l*, |
|  |  | int | *cur\_c* |
|  | ) |  |  |

Function to determine id a cell of the score array has to be evaluated after mutating two sequences.

Parameters
:   |  |  |
    | --- | --- |
    | mut1 | number of the first sequence mutated |
    | mut2 | number of the second sequence mutated |
    | cur\_l | current line of the array |
    | cur\_c | current column of the array |

Returns
:   0 if current cell doesn't need to be evaluated, 1 if it does

|  |  |  |  |  |  |
| --- | --- | --- | --- | --- | --- |
| int ck\_GC | ( | char \* | *DNA* | ) |  |

Function to check if GC-content is within a range.

Parameters
:   |  |  |
    | --- | --- |
    | DNA | Pointer to DNA sequence |

Returns
:   1 if GC-content is within range, 0 otherwise

See Also
:   GC
:   GC\_VAR

|  |  |  |  |  |  |
| --- | --- | --- | --- | --- | --- |
| int ck\_hairpin | ( | char \* | *cur\_struct* | ) |  |

Function to check if a DNA strand has hairpin.

Parameters
:   |  |  |
    | --- | --- |
    | cur\_struct | Pointer to the folded structure of the DNA sequence |

Returns
:   1 if less than MAX\_HAIRPIN of the strand is folded and 0 otherwise

See Also
:   MAX\_HAIRPIN

|  |  |  |  |  |  |
| --- | --- | --- | --- | --- | --- |
| int ck\_nt\_bias | ( | char \* | *DNA* | ) |  |

Function to check if single nucleotide bias is within a range.

Parameters
:   |  |  |
    | --- | --- |
    | DNA | Pointer to DNA sequence |

Returns
:   1 if single nucleotide bias is within range, 0 otherwise

See Also
:   NT
:   NT\_VAR

|  |  |  |  |
| --- | --- | --- | --- |
| int ck\_sec\_struct | ( | char \* | *cur\_struct*, |
|  |  | int | *where* |
|  | ) |  |  |

Function to check if a DNA strand has free ends.

Parameters
:   |  |  |
    | --- | --- |
    | cur\_struct | Pointer to the folded structure of the DNA sequence |
    | where | To tell which end has to be free. 1 for 5', 2 for 3', 3 for both |

Returns
:   1 if ends are free, 0 otherwise


---

Generated on Mon May 12 2014 15:06:53 for generate\_PLA\_lib by  

 1.8.6
