# Supplementary figures and images for: Design and Validation of DNA Libraries for Multiplexing Proximity Ligation Assays
Source: PLoS One. 2014 Nov 11;9(11):e112629. doi: 10.1371/journal.pone.0112629 (PMC4227721; doi:10.1371/journal.pone.0112629)

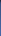

Supplement: File S1 — Source code of the program to generate PLA templates following the approach given in figure 2. Help and annotation notes are given in the file. (ZIP) [file pone.0112629.s002.zip › generate_PLA_lib/doc/html/tab_a.png]

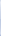

Supplement: File S1 — Source code of the program to generate PLA templates following the approach given in figure 2. Help and annotation notes are given in the file. (ZIP) [file pone.0112629.s002.zip › generate_PLA_lib/doc/html/tab_b.png]

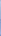

Supplement: File S1 — Source code of the program to generate PLA templates following the approach given in figure 2. Help and annotation notes are given in the file. (ZIP) [file pone.0112629.s002.zip › generate_PLA_lib/doc/html/tab_h.png]

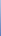

Supplement: File S1 — Source code of the program to generate PLA templates following the approach given in figure 2. Help and annotation notes are given in the file. (ZIP) [file pone.0112629.s002.zip › generate_PLA_lib/doc/html/tab_s.png]

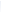

Supplement: File S1 — Source code of the program to generate PLA templates following the approach given in figure 2. Help and annotation notes are given in the file. (ZIP) [file pone.0112629.s002.zip › generate_PLA_lib/doc/html/nav_h.png]

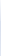

Supplement: File S1 — Source code of the program to generate PLA templates following the approach given in figure 2. Help and annotation notes are given in the file. (ZIP) [file pone.0112629.s002.zip › generate_PLA_lib/doc/html/nav_f.png]

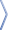

Supplement: File S1 — Source code of the program to generate PLA templates following the approach given in figure 2. Help and annotation notes are given in the file. (ZIP) [file pone.0112629.s002.zip › generate_PLA_lib/doc/html/bc_s.png]

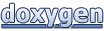

Supplement: File S1 — Source code of the program to generate PLA templates following the approach given in figure 2. Help and annotation notes are given in the file. (ZIP) [file pone.0112629.s002.zip › generate_PLA_lib/doc/html/doxygen.png]

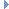

Supplement: File S1 — Source code of the program to generate PLA templates following the approach given in figure 2. Help and annotation notes are given in the file. (ZIP) [file pone.0112629.s002.zip › generate_PLA_lib/doc/html/closed.png]

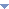

Supplement: File S1 — Source code of the program to generate PLA templates following the approach given in figure 2. Help and annotation notes are given in the file. (ZIP) [file pone.0112629.s002.zip › generate_PLA_lib/doc/html/open.png]

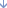

Supplement: File S1 — Source code of the program to generate PLA templates following the approach given in figure 2. Help and annotation notes are given in the file. (ZIP) [file pone.0112629.s002.zip › generate_PLA_lib/doc/html/bdwn.png]

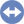

Supplement: File S1 — Source code of the program to generate PLA templates following the approach given in figure 2. Help and annotation notes are given in the file. (ZIP) [file pone.0112629.s002.zip › generate_PLA_lib/doc/html/sync_on.png]

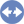

Supplement: File S1 — Source code of the program to generate PLA templates following the approach given in figure 2. Help and annotation notes are given in the file. (ZIP) [file pone.0112629.s002.zip › generate_PLA_lib/doc/html/sync_off.png]

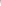

Supplement: File S1 — Source code of the program to generate PLA templates following the approach given in figure 2. Help and annotation notes are given in the file. (ZIP) [file pone.0112629.s002.zip › generate_PLA_lib/doc/html/nav_g.png]

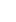

Supplement: File S1 — Source code of the program to generate PLA templates following the approach given in figure 2. Help and annotation notes are given in the file. (ZIP) [file pone.0112629.s002.zip › generate_PLA_lib/doc/html/ftv2blank.png]

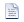

Supplement: File S1 — Source code of the program to generate PLA templates following the approach given in figure 2. Help and annotation notes are given in the file. (ZIP) [file pone.0112629.s002.zip › generate_PLA_lib/doc/html/ftv2doc.png]

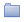

Supplement: File S1 — Source code of the program to generate PLA templates following the approach given in figure 2. Help and annotation notes are given in the file. (ZIP) [file pone.0112629.s002.zip › generate_PLA_lib/doc/html/ftv2folderclosed.png]

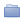

Supplement: File S1 — Source code of the program to generate PLA templates following the approach given in figure 2. Help and annotation notes are given in the file. (ZIP) [file pone.0112629.s002.zip › generate_PLA_lib/doc/html/ftv2folderopen.png]

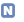

Supplement: File S1 — Source code of the program to generate PLA templates following the approach given in figure 2. Help and annotation notes are given in the file. (ZIP) [file pone.0112629.s002.zip › generate_PLA_lib/doc/html/ftv2ns.png]

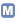

Supplement: File S1 — Source code of the program to generate PLA templates following the approach given in figure 2. Help and annotation notes are given in the file. (ZIP) [file pone.0112629.s002.zip › generate_PLA_lib/doc/html/ftv2mo.png]

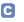

Supplement: File S1 — Source code of the program to generate PLA templates following the approach given in figure 2. Help and annotation notes are given in the file. (ZIP) [file pone.0112629.s002.zip › generate_PLA_lib/doc/html/ftv2cl.png]

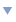

Supplement: File S1 — Source code of the program to generate PLA templates following the approach given in figure 2. Help and annotation notes are given in the file. (ZIP) [file pone.0112629.s002.zip › generate_PLA_lib/doc/html/ftv2mlastnode.png]

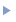

Supplement: File S1 — Source code of the program to generate PLA templates following the approach given in figure 2. Help and annotation notes are given in the file. (ZIP) [file pone.0112629.s002.zip › generate_PLA_lib/doc/html/ftv2plastnode.png]

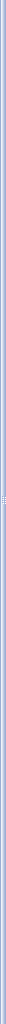

Supplement: File S1 — Source code of the program to generate PLA templates following the approach given in figure 2. Help and annotation notes are given in the file. (ZIP) [file pone.0112629.s002.zip › generate_PLA_lib/doc/html/ftv2splitbar.png]

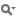

Supplement: File S1 — Source code of the program to generate PLA templates following the approach given in figure 2. Help and annotation notes are given in the file. (ZIP) [file pone.0112629.s002.zip › generate_PLA_lib/doc/html/search/mag_sel.png]

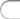

Supplement: File S1 — Source code of the program to generate PLA templates following the approach given in figure 2. Help and annotation notes are given in the file. (ZIP) [file pone.0112629.s002.zip › generate_PLA_lib/doc/html/search/search_l.png]

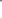

Supplement: File S1 — Source code of the program to generate PLA templates following the approach given in figure 2. Help and annotation notes are given in the file. (ZIP) [file pone.0112629.s002.zip › generate_PLA_lib/doc/html/search/search_m.png]

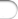

Supplement: File S1 — Source code of the program to generate PLA templates following the approach given in figure 2. Help and annotation notes are given in the file. (ZIP) [file pone.0112629.s002.zip › generate_PLA_lib/doc/html/search/search_r.png]

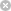

Supplement: File S1 — Source code of the program to generate PLA templates following the approach given in figure 2. Help and annotation notes are given in the file. (ZIP) [file pone.0112629.s002.zip › generate_PLA_lib/doc/html/search/close.png]

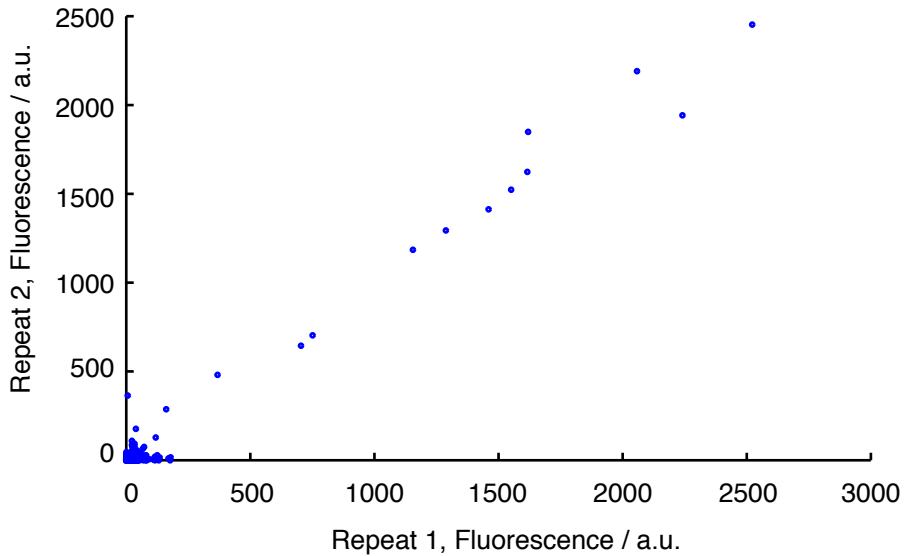

Supplement: File S4 — Chip-to-chip correlation for the spRCA reactions. The graph shows the correlation of the spRCA fluorescence signals between repeats for the complete spRCA library screen. Repeats were performed within different chip runs. (PDF) [file pone.0112629.s005.pdf]
