# Supplementary material for: Design and Validation of DNA Libraries for Multiplexing Proximity Ligation Assays
Source: PLoS One. 2014 Nov 11;9(11):e112629. doi: 10.1371/journal.pone.0112629 (PMC4227721; doi:10.1371/journal.pone.0112629)
Supplement: File S2 — Microfluidic workflow of the solid phase RCA with a chemical reagent list. (PDF) [file pone.0112629.s003.pdf]

File S1. Microfluidic workflow of the solid phase RCA with a chemical reagent list. Note, in detailed description of the microlfuidic chip design and function is given in references [4, 21].

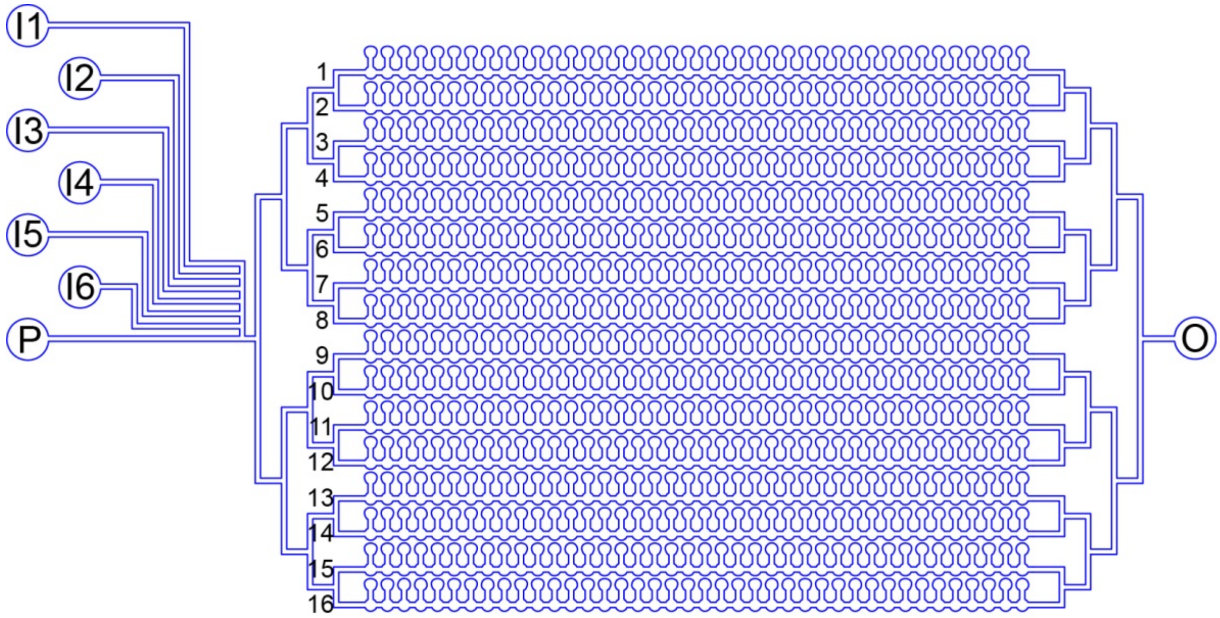

The above figure shows the fluidic layer of the microfluidic chip used for the spRCA and spPLA. Reagents inlet ports, row elements with 40 unit cells, and the outlet port are numbered. Description of the fluid routing at each proecess step in the table below follows the numbering. For example: Biotinylated BSA is introduced from inlet port 1 (I1), is flushed through all rows of the chip (1-16), to the outlet (O).

Section	#	Step	Sec	T°	From	To	Lines	Button
Surface Chemistry	1	Flush Device with Biotinylated BSA	750	RT	I2	O	1->16	open
	2	Purge Inlet with PBS 1x	30		I1	P		
	3	Flush Device with PBS 1x	750	RT	I1	O	1->16	open
	4	Purge Inlet with NeutrAvidin	30		I3	P		
	5	Flush Device with NeutrAvidin	750	RT	I3	O	1->16	open
	6	Purge Inlet with PBS 1x	30		I1	P		
	7	Flush Device with PBS 1x	750	RT	I1	O	1->16	open
	8	Close Buttons	120					close
	9	Purge Inlet with Biotinylated BSA	30		I2	P		
	10	Flush Device with Biotinylated BSA	750	RT	I2	O	1->16	close
	11	Purge Inlet with PBS 1x	30		I1	P		
	12	Flush Device with PBS 1x	750	RT	I1	O	1->16	close
DNA Pull-down	13	Fill back-chambers	120	RT	I1		1->16	close
	14	Purge Inlet with PBS 1x	30		I1	P		
	15	Flush Device with PBS 1x	750	RT	I1	O	1->16	close
	16	Separate chambers	20					
	17	Open back-chambers	30					
	18	Open button and incubate	600					open
	19	Close button	60					close
	20	Purge Inlet with PBS 1x	30		I1	P		
	21	Flush Device with PBS 1x	600	RT	I1	O	1->16	close
Ligation	22	Wash inlet I2	60		I1	I2		
	23	Wash inlet I3	60		I1	I3		
	24	Purge Inlet with ligation 1	30		I2	P		
	25	Flush Device with ligation 1	750	40°C	I2	O	1->4	close
	26	Purge Inlet with ligation 2	30	40°C	I3	P		
	27	Flush Device with ligation 2	750	40°C	I3	O	5->8	close
	28	Purge Inlet with ligation 3	30	40°C	I4	P		
	29	Flush Device with ligation 3	750	40°C	I4	O	9->12	close
	30	Purge Inlet with ligation 4	30	40°C	I5	P		
	31	Flush Device with ligation 4	750	40°C	I5	O	13->16	close
	32	Open button and incubate	900	40°C				open
	33	Close button	60	40°C				close
	34	Purge Inlet with PBS 1x	30		I1	P		
	35	Flush Device with PBS 1x	600	RT	I1	O	1->16	close
RCA	36	Open Button	10					open
	37	Purge Inlet with RCA	30		I6	P		
	38	Flush Device with RCA	900	32°C	I6	O	1->16	open
	39	Incubate RCA	7200	32°C				open
	40	Purge Inlet with PBS 1x	30		I1	P		
	41	Flush Device with PBS 1x	900	RT	I1	O	1->16	open
Dye	42	Wash inlet I4	60		I1	I4		
	43	Flush Device with Dye	1200	30°C	I4	O	1->16	open
	44	Purge Inlet with PBS 1x	30		I1	P		
	45	Flush Device with PBS 1x	900	RT	I1	O	1->16	open

Biotinylated BSA	0,5mg/ml biotinylated bovine serum albumin (Thermo Scientific) in 1xPBS
NeutrAvidin	0,5mg/ml NeutrAvidin (Thermo Scientific) in 1xPBS
Ligation mix	500 mM of each connector in 40 mM Tris-HCl, 10 mM MgCl2, 10 mM DTT, 5 mM ATP and 0,2 U/µl T4 DNA ligase (Fermentas)
RCA	0.125 U/µl phi29 polymerase (New England Biolabs) with 250 µM dNTP and 0.2 mg/ml BSA in 5mM Tris-HCl, 1mM $MgCl_2$, 1mM $(NH_4)_2SO_4$, 400µM DTT buffer
Dye	1µM probe labeled with [6FAM] (Sigma) in 1x SSC
