# Supplementary material for: Design and Validation of DNA Libraries for Multiplexing Proximity Ligation Assays
Source: PLoS One. 2014 Nov 11;9(11):e112629. doi: 10.1371/journal.pone.0112629 (PMC4227721; doi:10.1371/journal.pone.0112629)
Supplement: File S3 — Microfluidic workflow of the solid phase PLA with a chemical reagent list. (PDF) [file pone.0112629.s004.pdf]

File S2. Microfluidic workflow of the solid phase PLA with a chemical reagent list.

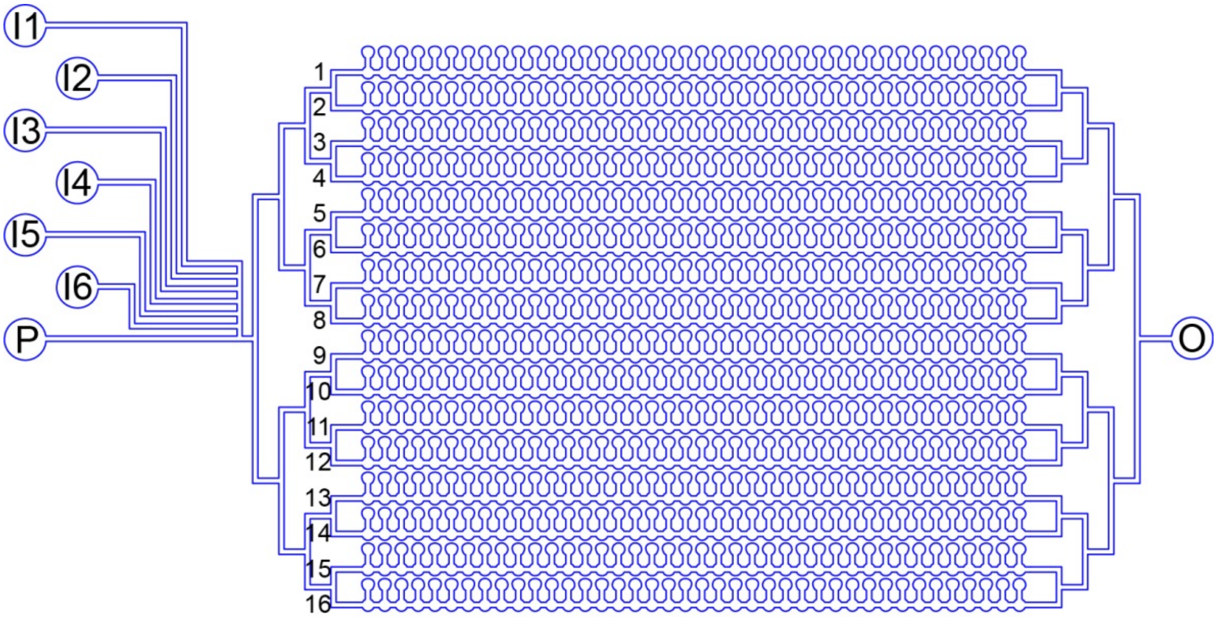

The above figure shows the fluidic layer of the microfluidic chip used for the spRCA and spPLA. Reagents inlet ports, row elements with 40 unit cells, and the outlet port are numbered. Description of the fluid routing at each proecess step in the table below follows the numbering. For example: Biotinylated BSA is introduced from inlet port 1 (I1), is flushed through all rows of the chip (1-16), to the outlet (O).

		Step	Sec	T°	From	To	Lines	Button
Surface Chemistry	1	Flush Biotinylated BSA	750	RT	I2	O	1->16	open
	2	Purge Inlet with PBS 1x	30		I1	P		
	3	Flush Device with PBS 1x	750	RT	I1	O	1->16	open
	4	Purge Inlet with NeutrAvidin	30		I3	P		
	5	Flush Device with NeutrAvidin	750	RT	I3	O	1->16	open
	6	Purge Inlet with PBS 1x	30		I1	P		
	7	Flush Device with PBS 1x	750	RT	I1	O	1->16	open
	8	Close Buttons	120					close
	9	Purge Inlet with Biotinylated BSA	30		I2	P		
	10	Flush Device with Biotinylated BSA	750	RT	I2	O	1->16	close
	11	Purge Inlet with PBS 1x	30		I1	P		
	12	Flush Device with PBS 1x	750	RT	I1	O	1->16	close
biotin sAB	13	Wash inlet I2	60		I1	I2		
	14	Wash inlet I3	60		I1	I3		
	15	Purge inlet with biotinylated AB anti-goat	60		I4	P		
	16	Flush Device with biotinylated AB anti-goat	750	RT	I4	O	1->16	open
	17	Purge Inlet with PBS 1x	30		I1	P		
	18	Flush Device with PBS 1x	500	RT	I1	O	1->16	open
pAB-1 pulldown	19	Purge inlet with first conjugated AB1 anti-VEGF	30		I5	P		
	20	Flush Device with first conjugated AB1 anti-VEGF	1000	RT	I5	O	1->8	open
	21	Purge Inlet with PBS 1x	30		I1	P		
	22	Flush Device with PBS 1x	500	RT	I1	O	1->8	open
	23	Purge inlet with second conjugated AB1 anti-VEGF	30		I6	P		
	24	Flush Device with second conjugated AB1 anti-VEGF	1000	RT	I6	O	9->16	open
	25	Purge Inlet with PBS 1x	30		I1	P		
	26	Flush Device with PBS 1x	500	RT	I1	O	9->16	open
Analyte	27	Wash inlet I4	60		I1	I4		
	28	Wash inlet I5	60		I1	I5		
	29	Wash inlet I6	60		I1	I6		
	30	Purge inlet with human VEGF121	30		I2	P		
	31	Flush Device with human VEGF121	1000	RT	I2	O	1->16	open
	32	Purge Inlet with PBS 1x	30	RT	I1	P		
	33	Flush Device with PBS 1x	500	RT	I1	O	1->16	open
pAB-2 pulldown	34	Purge inlet with 1st conjugated AB2 anti-VEGF (from rabbit)	30		I3	P		
	35	Flush Device with 1st conjugated AB2 anti-VEGF (from rabbit)	500	RT	I3	O	1	open
	36	Purge inlet with 2nd conjugated AB2 anti-VEGF (from rabbit)	30		I4	P		
	37	Flush Device with 2nd conjugated AB2 anti-VEGF (from rabbit)	500	RT	I4	O	2	open
	38	Purge inlet with 3rd conjugated AB2 anti-VEGF (from rabbit)	30		I5	P		
	39	Flush Device with 3rd conjugated AB2 anti-VEGF (from rabbit)	500	RT	I5	O	3	open
	40	Purge inlet with 4th conjugated AB2 anti-VEGF (from rabbit)	30		I6	P		
	41	Flush Device with 4th conjugated AB2 anti-VEGF (from rabbit)	500	RT	I6	O	4	open
	42	Purge Inlet with PBS 1x	30		I1	P		
	43	Flush Device with PBS 1x	500	RT	I1	O	1->16	open
	44	Wash inlet I3	60		I1	I3		
	45	Wash inlet I4	60		I1	I4		
	46	Wash inlet I5	60		I1	I5		
	47	Wash inlet I6	60		I1	I6		
	48	Purge inlet with 5th conjugated AB2 anti-VEGF (from rabbit)	30		I3	P		

	49	Flush Device with 5th conjugated AB2 anti-VEGF (from rabbit)	500	RT	I3	O	5	open
	50	Purge inlet with 6th conjugated AB2 anti-VEGF (from rabbit)	30		I4	P		
	51	Flush Device with 6th conjugated AB2 anti-VEGF (from rabbit)	500	RT	I4	O	6	open
	52	Purge inlet with 7th conjugated AB2 anti-VEGF (from rabbit)	30		I5	P		
	53	Flush Device with 7th conjugated AB2 anti-VEGF (from rabbit)	500	RT	I5	O	7	open
	54	Purge inlet with 8th conjugated AB2 anti-VEGF (from rabbit)	30		I6	P		
	55	Flush Device with 8th conjugated AB2 anti-VEGF (from rabbit)	500	RT	I6	O	8	open
	56	Purge Inlet with PBS 1x	30		I1	P		
	57	Flush Device with PBS 1x	500	RT	I1	O	1->16	open
ligation	58	Wash inlet I2	60		I1	I2		
	59	Purge Inlet with Ligation	30		I2	P		
	60	Flush Device with Ligation	750	40°(	I2	O	1->16	close
	61	Fill back-chambers	120	40°(	I2			close
	62	Separate chambers	20	40°C				close
	63	Open button and incubate	900	40°C				open
	64	Close button	60	40°C				close
	65	Purge Inlet with PBS 1x	30		I1	P		
	66	Flush Device with PBS 1x	600	RT	I1	O	1->16	close
RCA	67	Wash inlet I3	60		I1	I3		
	68	Open Button	10					open
	69	Purge Inlet with RCA	60	RT	I1	I3		
	70	Flush Device with RCA	900	32°(	I3	O	1->16	open
	71	Incubate RCA	7200	32°C				open
	72	Purge Inlet with PBS 1x	30		I1	P		
	73	Flush Device with PBS 1x	900	RT	I1	O	1->16	open
Dye	74	Wash inlet I4	60		I1	I4		
	75	Flush Device with Dye	1200	30°(	I4	O	1->16	open
	76	Purge Inlet with PBS 1x	30		I1	P		
	77	Flush Device with PBS 1x	900	RT	I1	O	1->16	open

Biotinylated BSA	0,5mg/ml biotinylated bovine serum albumin (Thermo Scientific) in 1xPBS
NeutrAvidin	0,5mg/ml NeutrAvidin (Thermo Scientific) in 1xPBS
Biotinylated AB anti-goat	40ng/µl Anti-Goat IgG (whole molecule)-Biotin (Sigma) in 1x PBS
Conjugated Adaptor-1 anti-VEGF	6ng/µl adaptor1-conjugated anti-hVEGF165 IgG produced in goat (R&D Systems) in 1x PBS 0,1% BSA
Human VEGF165	10ng/µl recombinant human VEGF165 (R&D Systems) in 1X PBS
Conjugated Adaptor-2 anti-VEGF	4ng/µl adaptor2-conjugated anti-hVEGF IgG produced in rabbit (Thermo Scientific) in 1x PBS 0,1% BSA
Ligation mix	500 mM of each connector in 40 mM Tris-HCl, 10 mM MgCl2, 10 mM DTT, 5 mM ATP and 0,2 U/µl T4 DNA ligase (Fermentas)
RCA	0.125 U/µl phi29 polymerase (New England Biolabs) with 250 µM dNTP and 0.2 mg/ml BSA in 5mM Tris-HCl, 1mM $MgCl_2$, 1mM $(NH_4)_2SO_4$, 400µM DTT buffer
Dye	1µM probe labeled with [6FAM] (Sigma) in 1x SSC
